# Novel insight into the spatiotemporal distribution of Greenland ice sheet surface densities from eleven years of satellite radar altimetry

**DOI:** 10.1038/s41598-025-02403-2

**Published:** 2025-05-17

**Authors:** Kirk M. Scanlan, Anja Rutishauser, Nicolaj Hansen, Sebastian B. Simonsen

**Affiliations:** 1 DTU Space, Technical University of Denmark, Kgs. Lyngby, 2800 Denmark; 2 Department of Glaciology and Climate, Geological Survey of Denmark and Greenland, Copenhagen, 1350 Denmark; 3 Department of National Centre for Climate Research (NCKF), Danish Meteorological Institute, Copenhagen, 2100 Denmark

**Keywords:** Cryospheric science, Climate change

## Abstract

Greenland Ice Sheet near-surface density is a key parameter as it facilitates converting changes in volume from repeat satellite altimetry to changes in mass. However, the need to continually quantify density through both space and time means it is conventionally derived using numerical models. Recently, new focus is being placed on directly deriving ice sheet surface properties from satellite observations. Here we show how 11 years of surface densities derived from ESA CryoSat-2 and CNES/ISRO SARAL radar altimetry compare against in situ and regional climate model results. We find that a consistent interpretation of the satellite near-surface densities is strongly modulated by the presence of melt. For periods when the ice sheet surface can be considered frozen, satellite-based densities best represent in situ conditions at different intervals in the top 1.25 m. At the same time, while long-term pan-Greenland observation- and model-based mean densities are similar, the observations reveal spatial patterns absent in the models and consistently greater densities in the ice sheet interior. In months with strong melting, the satellite results return anomalously low surface densities, though accounting for associated changes in radar backscattering phenomena and stratigraphic interpretation are limited by the lack of contemporaneous in situ data. Overall, we establish a quantitative approach for interpreting novel densities derived exclusively from satellite observations and present a comparison to state-of-the-art model results.

**Supplementary Information:**

The online version contains supplementary material available at 10.1038/s41598-025-02403-2.

For the firn-covered portion of the Greenland Ice Sheet (GrIS), near-surface density facilitates deriving mass budgets from long-term satellite radar altimetry volume changes^1–7^ and is conventionally assessed using climate and firn densification models^8–16^. Leveraging a method (i.e., Radar Statistical Reconnaissance; RSR) initially developed to study the surface of Mars^17,18^, but that has also been applied to airborne 60 MHz very-high frequency (VHF) radar measurements^19–22^, Scanlan et al.^23^ produced the first set of maps and a six-year (i.e., 2013–2018) monthly timeseries of GrIS near-surface density derived exclusively from CNES/ISRO SARAL^24,25^ and ESA CryoSat-2^26–28^ radar altimetry measurements. However, this initial study focused on outlining the procedure for generating these results and performing a preliminary but limited qualitative interpretation. Here we present a longer, eleven-year (2013–2023) timeseries of near-surface density for the > 1500 m elevation portion of the GrIS (i.e., 1500 m is assumed to represent the snowline elevation marking the boundary between seasonal snow cover and firn) and perform quantitative comparisons against (a) a community compilation of in situ density measurements^29^ (from surface samples, snow pits, firn cores, borehole logs etc.) and (b) near-surface densities from state-of-the-art regional climate models (RCMs); v3.14 of MAR^30,31^ and HIRHAM5^11,32,33^.

## Results

### Comparison to in situ densities

**Fig. 1:**
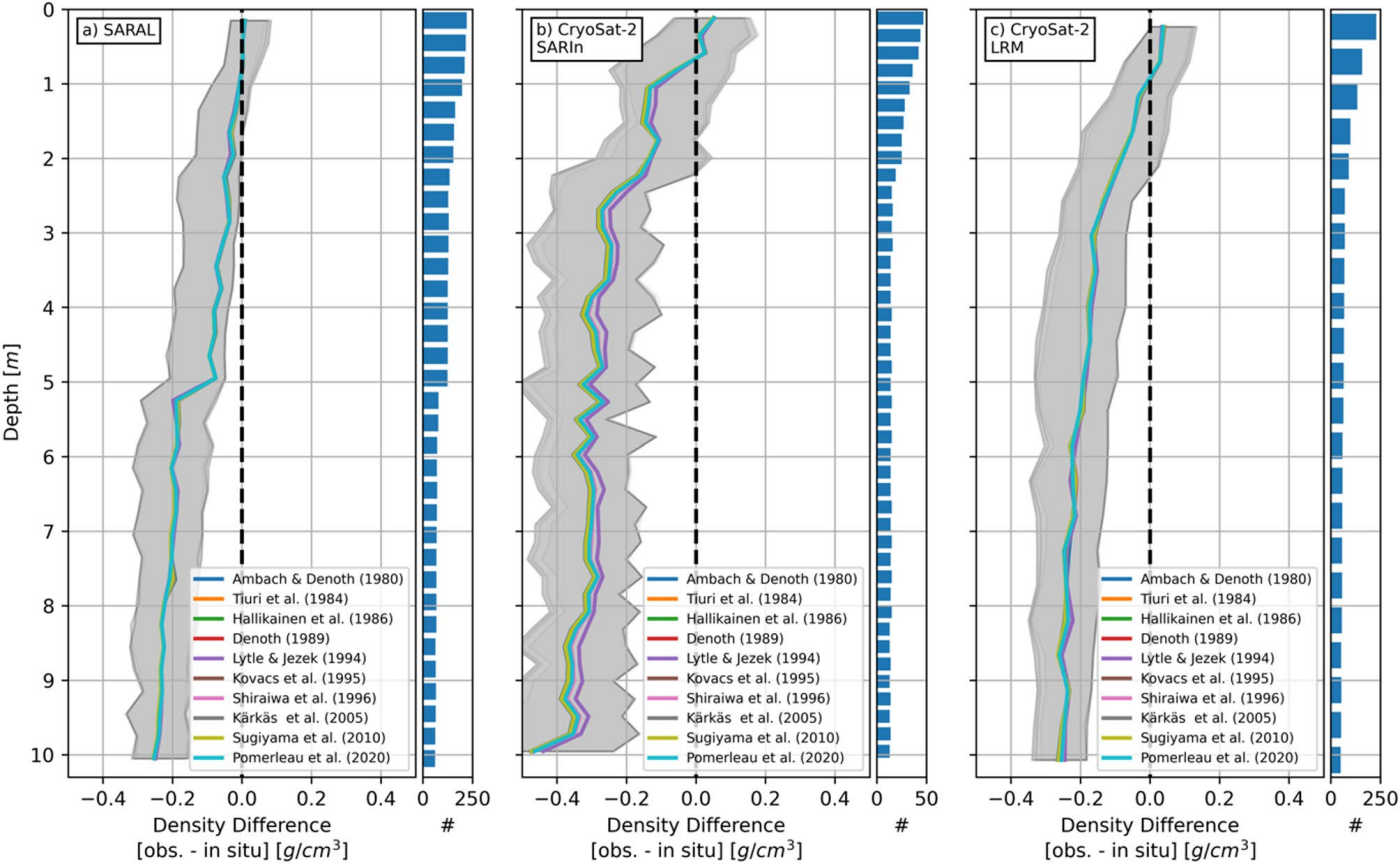
Comparison of (**a**) SARAL, (**b**) CryoSat-2 SARIn, and (**c**) CryoSat-2 LRM densities against in situ measurements. Shaded areas denote the interquartile range. Radar-specific representative depths are defined where the median density difference is zero. Bar plots represent the number of radar-to-model density comparisons within each depth increment. Results for the individual dielectric permittivity-to-density conversion models are presented in the Supplementary Materials.

Based on the observed density magnitudes, the initial interpretation^23^ concluded that the lower-frequency Ku-band (13.575 GHz) ESA CryoSat-2 density estimates represent a deeper portion of the GrIS near-surface compared to those derived from Ka-band (35.75 GHz) CNES/ISRO SARAL measurements. Figure 1 presents the results of a more quantitative approach, comparing altimetry-derived densities against contemporaneous (i.e., same month) resampled (see Online Methods for resampling procedure) in situ^29^ measurements above the GrIS snowline (herein defined as the 1500 m elevation contour). The representative depth of the altimetry-derived density measurement is taken where the median density difference is zero and the associated uncertainty is determined by the interquartile range. The horizontal bar plots denote the total number of observation/in situ comparisons at each depth interval for that specific radar dataset.

The representative depth intervals and uncertainties for densities derived from SARAL (Fig. 1a), CryoSat-2 Synthetic Aperture Radar Interferometric (SARIn) (Fig. 1b), and CryoSat-2 Low-Resolution Mode (LRM) (Fig. 1c) surface echo powers are 0–1.0 m and ±0.06 g/cm^3^, 0.5–0.8 m and ±0.13 g/cm^3^, and 0.75–1.25 m and ±0.08 g/cm^3^, respectively. These results are not strongly sensitive to the models^34–43^ used to convert the RSR-derived relative dielectric permittivities to densities. The CryoSat-2 SARIn results do exhibit more model-dependent variability but also the fewest points of comparison, which contributes to the larger uncertainty. The step change in the median SARAL density difference at five meters depth appears related to a sharp decrease in the number of comparative in situ measurements.

These results suggest that the joint analysis of dual-frequency radar altimetry measurements and their relative densities can shed light on the vertical density structure and stratigraphy of the GrIS. The along-track processing applied to CryoSat-2 LRM waveforms (20 Hz waveform stacking) and coarser waveform sampling (0.4684 m for CryoSat-2 LRM compared to 0.2342 m for CryoSat-2 SARIn) likely contributes to the deeper CryoSat-2 LRM representative depths. Even though SARAL waveforms are subject to similar along-track processing and have a coarser waveform sampling than CryoSat-2 SARIn, the higher Ka-band frequency keeps SARAL more sensitive to the upper portion of the GrIS. Looking forward, this presents a unique opportunity for retrieving GrIS near-surface stratigraphy from future dual frequency Ku-/Ka-band altimeters such as onboard ESA’s Copernicus Polar Ice and Snow Topography Altimeter (CRISTAL) spacecraft.

### Long-term comparison to model densities

**Fig. 2:**
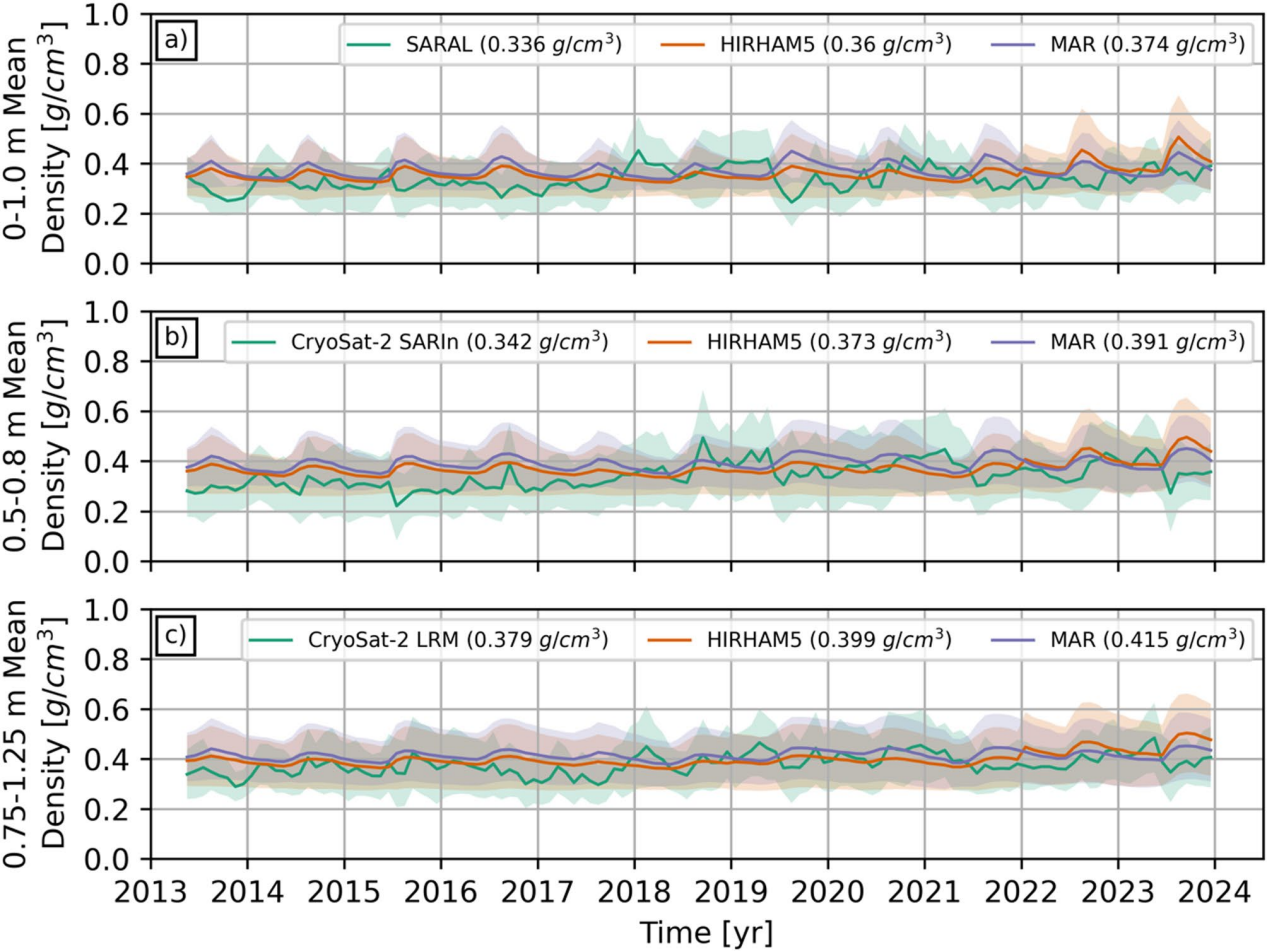
Long-term observation and model mean GrIS density timeseries for the three representative depth intervals corresponding to the (**a**) SARAL (0–1.0 m), (**b**) CryoSat-2 SARIn (0.5–0.8 m), and (**c**) CryoSat-2 LRM (0.75–1.25 m) radar datasets. Shaded area around the mean timeseries represents one standard deviation in reported densities for that month. The 2013–2023 means are given in parentheses in the individual legends. Long-term mean timeseries are generally consistent, while HIRHAM5 and MAR exhibit distinct seasonal patterns.

With representative depth intervals for the three different sets of radar altimetry surface densities, they are now compared against similar depth-resolved results from MAR^30,31^ and HIRHAM5^11,32,33^. RCM- and observation-based density results are compiled at the same set of comparative locations (see Online Methods) and the mean density timeseries for the three depth intervals (i.e., SARAL, CryoSat-2 SARIn, and CryoSat-2 LRM) are shown in Fig. 2. The shaded areas represent one standard deviation in monthly pan-GrIS densities from each dataset (see Online Methods for how the monthly datasets are derived). The overall 2013–2023 means are given in parentheses in the individual sub-figure legends. Similar timeseries broken down into 500 m GrIS elevation intervals are presented in the Supplementary Materials.

How the radar-based densities compare to the RCM results is similar across the three representative depth intervals. Recognizing the overall substantial overlap between the timeseries, between 2013 and 2017 HIRHAM5 and MAR consistently return mean GrIS densities that are slightly larger than those derived from the analysis of surface echo powers. This difference is most obvious in the 0.5–0.8 m CryoSat-2 SARIn depth interval (Fig. 2b). The second half of the timeseries (i.e., 2018–2023) reveals a much closer agreement between the mean density values for all comparisons, though model densities remain slightly larger.

### Summer effects on radar-retrieved surface density

**Fig. 3:**
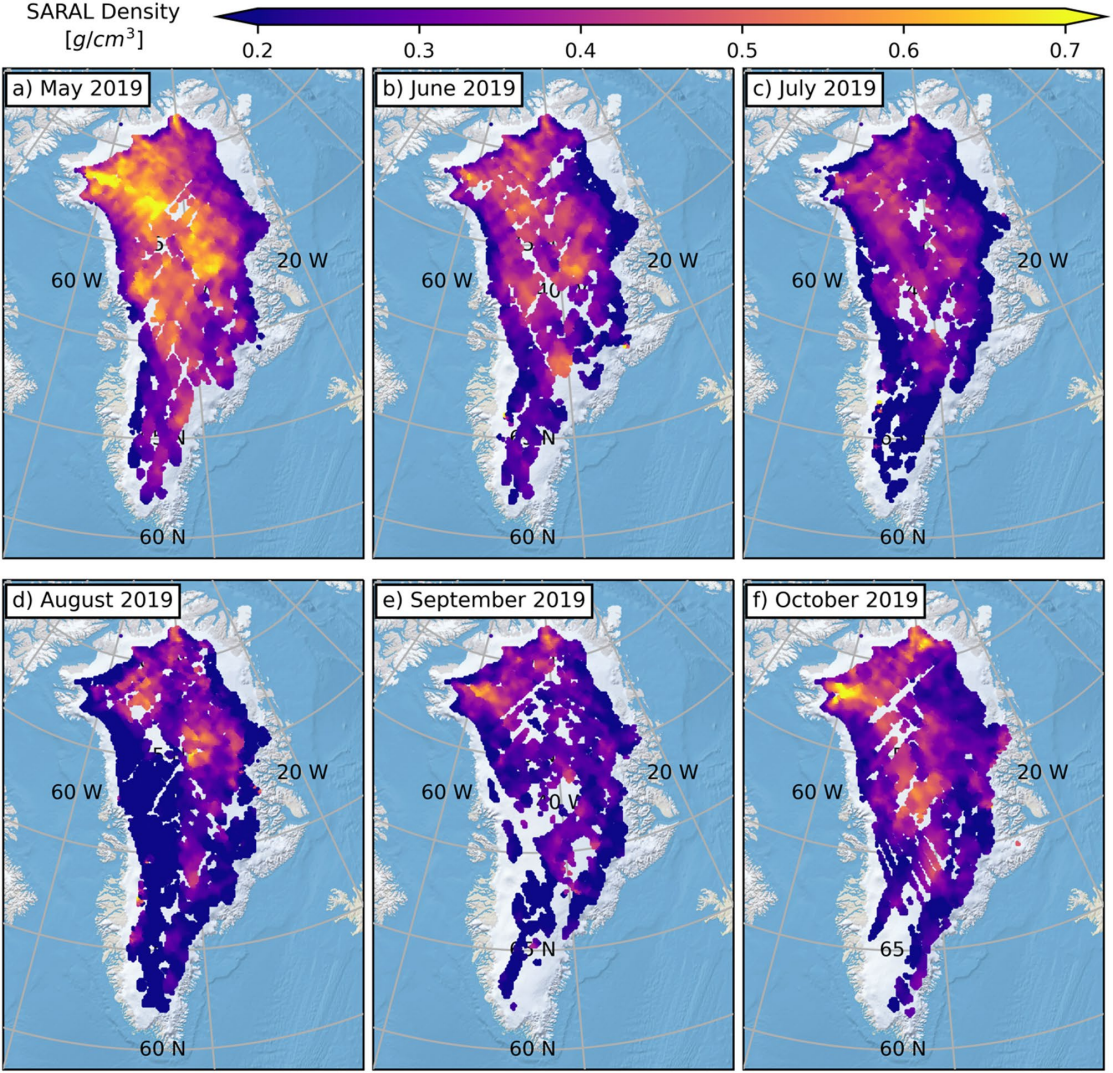
Monthly surface densities derived from SARAL measurements across the anomalous Summer 2019 melt season. Contrary to expectations, surface density decreases with increased surface melting. Gaps in the individual maps correspond to locations where low-quality RSR results have been removed (see Online Methods). This figure is made in Python3 (v3.8.8) using the public domain Natural Earth II basemap.

Common to all Fig. 2 timeseries, RCM results exhibit clear seasonal summer density peaks, while the observational results do not. In fact, summer months exhibit some of the lowest RSR-derived densities. The most distinct example is for SARAL (Fig. 2a) in 2019. Summer 2019 is known to have been abnormally warm, with two ~ 14-day long melt events; one in mid-June and another in late-July/early-August^31^. With 95.8% of the GrIS experiencing melt at some point in 2019, this melt season rivalled that of 2012^31^. The onset of melting in June 2019 corresponds to a progressive month-to-month reduction in SARAL densities from May until August (Fig. 3). Interestingly, while the reduction was generally pan-GrIS for May to July, the drop was most prominent in west Greenland between July and August (east Greenland densities are more stable). At their lowest point, SARAL densities in west Greenland are roughly 0.2 g/cm^3^. After the anomalous summer melt, SARAL-derived surface densities eventually increased to level consistent with the long-term mean (Figs. 2a and 3).

That melting would be accompanied by reduced surface densities is counterintuitive. At high GrIS elevations, in such an anomalous melt year, one could reasonably expect melt not to runoff, but to re-freeze, leading to densification. However, a small handful of melt days^31^ may not generate enough melt to substantially raise the densities within the vertically averaged depth intervals the radar signals are responding to (Fig. 1). Regardless, one would expect the density to remain roughly constant or marginally increase, not substantially drop. Radar-based GrIS surface densities are derived from backscattered surface echo powers, and the drop in density can be linked to weaker coherent surface reflections. Typically, the higher dielectric permittivity of liquid water would imply stronger reflection coefficients, but wet snow has been shown to have a lower normal incidence backscattering coefficient at Ku- and Ka-band frequencies compared to dry conditions^44,45^. This is likely due to the physical ways in which radar waves are reflected from a wet snow surface (e.g., enhancing the effects of surface roughness, reductions in the contribution from volume scattering) that are not being accounted for in the spatiotemporally-constant model used to invert RSR outputs for surface properties^17,20,23^. Muted versions of this phenomena may also explain other summer surface density drops in 2015 (Fig. 2b), 2016 (Fig. 2a), and 2023 (Fig. 2b).

### Summer effects on representative depths

Underlying Fig. 2 is the assumption that the representative depths (Fig. 1) are constant. In addition to altering radar signal backscattering from the GrIS, summer melting likely also affects the representative depths, as large (10’s cm) changes in the depth of the CryoSat-2 reflecting surface have been observed in response to large melt events^46^. The ability to characterize how the representative depths of the radar-derived densities change in response to summer GrIS climatic conditions though is hampered by the lack of contemporaneous in situ measurements to compare the satellite observations against (e.g., constructing a summer-only version of Fig. 1).

Of the 463 in situ measurements between May 2013 and December 2023 reported in the most recently community database^29^, 388 of them (83.8%) were collected in either April (149) or May (239). Even before further reducing the number of comparative in situ locations to only those exhibiting high-quality contemporaneous density observations (see Online Methods), the altimetry-based density representative depths are highly biased to spring conditions when the GrIS surface can considered to be frozen. Without in situ measurements, we cannot reliably establish how the representative depths are affected by summer melt. Constant representative depths represent the best quantification that can be done with the available data and considered representative of non-melt GrIS surface conditions. It should also be noted that spring-biased in situ data are also the foundation of the implemented parameterizations in the RCMs.

### Long-term Spatial patterns

**Fig. 4:**
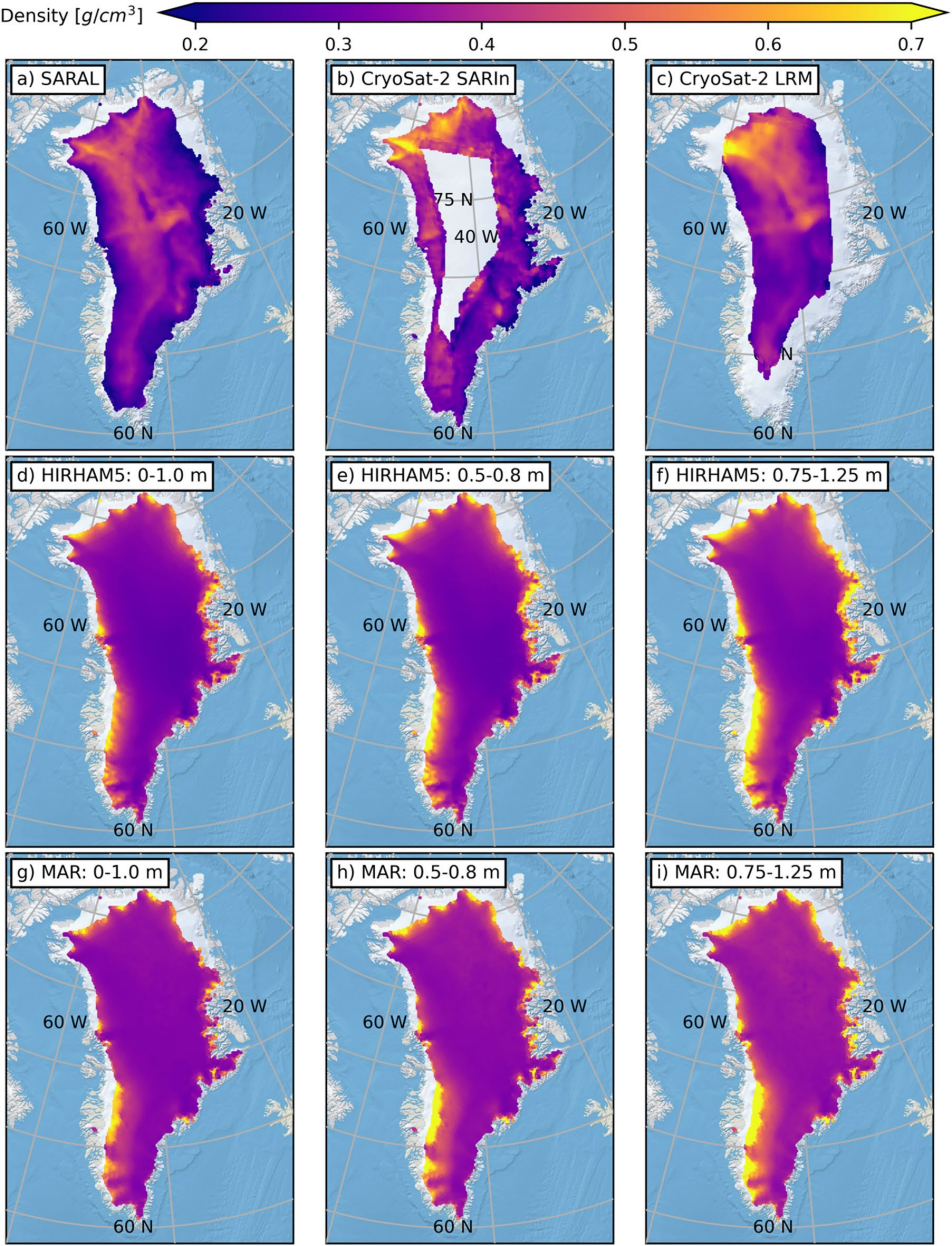
2013–2023 (JJA excluded) mean densities across the GrIS for (**a**) SARAL, (**b**) CryoSat-2 SARIn, (**c**) CryoSat-2 LRM, (**d**-**f**) HIRHAM5, and **g**)-**i**) MAR. RCM results are broken down by representative depth intervals. Variation in model densities occurs primarily around the margins while radar densities exhibit more variability across the entire GrIS. This figure is made in Python3 (v3.8.8) using the public domain Natural Earth II basemap.

**Fig. 5:**
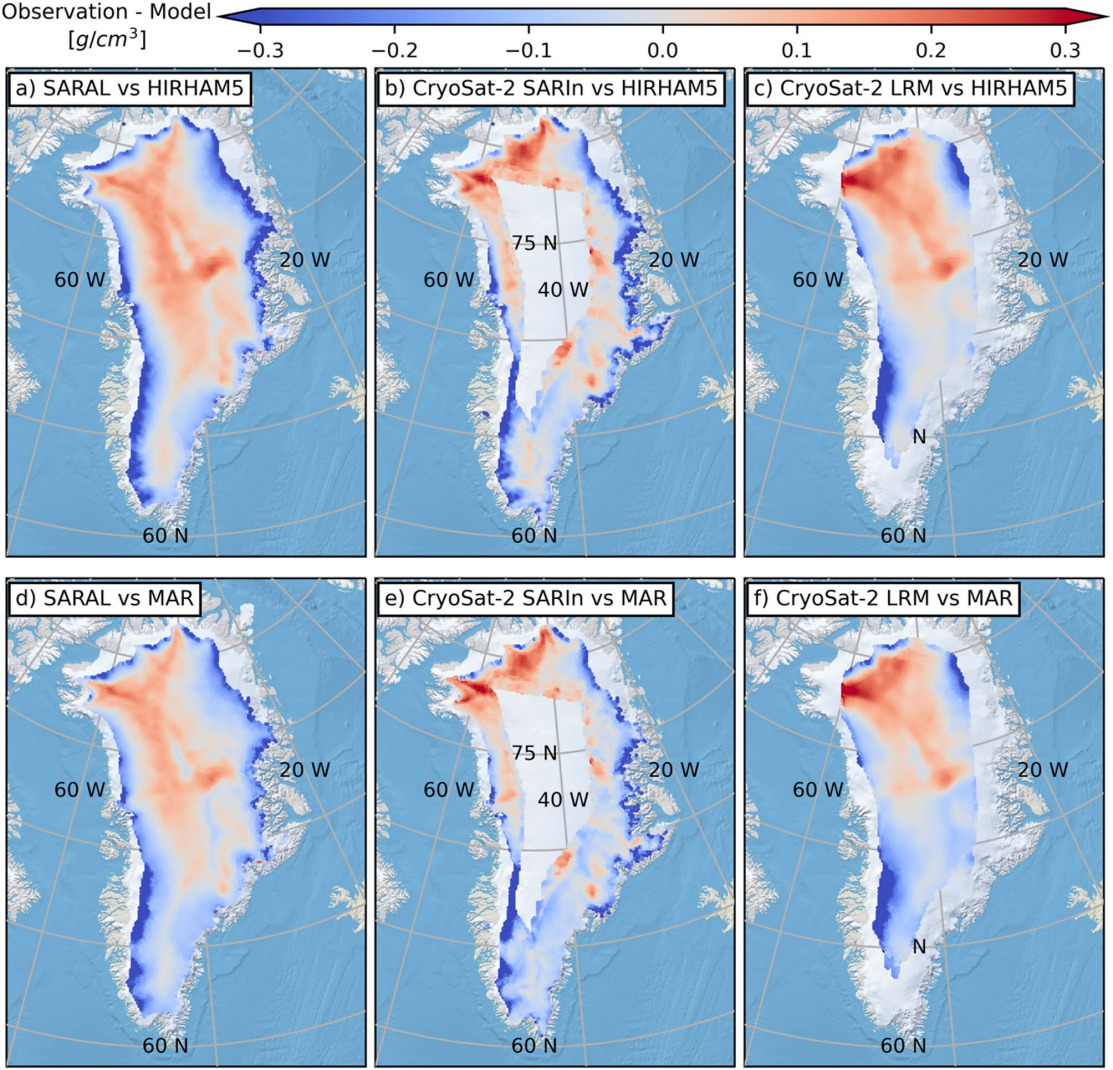
Differences in 2013–2023 (JJA excluded) mean density across the GrIS. Radar densities exceed those derived from the models in the ice sheet interior while the situation is reversed around the margin. This figure is made in Python3 (v3.8.8) using the public domain Natural Earth II basemap.

Overall, better characterization of summer (i.e., June, July, and August) GrIS in situ conditions, and specifically how they change the nature of SARAL and CryoSat-2 surface echoes is required to improve the reliability of the derived summer surface densities. In its absence, a complete year-round interpretation of the radar altimetry surface echo powers will remain elusive. Outside of summer months though, even though in situ measurements are heavily biased to April and May, inferences drawn from comparing the satellite results to in situ measurements are considered representative of non-melt conditions. In response, summer (e.g., JJA) densities are omitted from the long-term mean density maps in Fig. 4 and the density difference (radar minus RCM in the equivalent depth interval) maps in Fig. 5.

From Fig. 4, a clear benefit of the radar-derived densities is that they reveal much more complex spatial variations in surface density than the RCM results. All spatial variability in the HIRHAM5 (Figs. 4d-f) and MAR (Figs. 4g-i) surface densities occurs at low elevations; mean RCM densities in the ice sheet interior are effectively constant. In contrast, mean near-surface densities derived from SARAL (Fig. 4a) and CryoSat-2 (SARIn in Fig. 4b and LRM in Fig. 4c) tend to increase from ~ 0.25 g/cm^3^ just above the snowline to ~ 0.4–0.45 g/cm^3^ in the interior. Though there are exceptions such as southeast Greenland where lower surface densities persist into deeper (i.e., to higher elevation) portions of the ice sheet (Fig. 4a and b). Interestingly, all radar-based datasets return the highest surface densities in northwest Greenland and, while considering the different spatial radii over which surface echo powers are collected during RSR processing (see Online Methods), the spatial patterns are markedly consistent. Both SARAL and CryoSat-2 return sustained elevated densities in an area north of Summit (73.5°N, 37.4°W) and exhibit a band of lower densities just to the west (mainly stoss) side of the ice divide. The relative interplay of numerous climatic factors including winds, new snow accumulation, surface melt and internal refreezing likely contribute to the spatial complexity found in the radar-derived near-surface densities.

That the spatial patterns exhibited in the SARAL and CryoSat-2 mean density maps dominate the observation-to-RCM density difference maps (Fig. 5), speaks again to the homogeneity in RCM results. In the GrIS interior radar-based mean near-surface densities are roughly 0.1 g/cm^3^ greater than those returned by the model, but this increases in the regions with concentrated high elevations (e.g., northwest Greenland, north of Summit) to ≥0.2 g/cm^3^. The band of reduced radar-derived densities to the west of the central GrIS ice divide is roughly 0.05 g/cm^3^ lower than the RCM results (the difference is slightly larger for MAR than HIRHAM5). It is in the mid-elevations where the agreement between the RCM and radar results is best, though this does persist into the more marginal areas of the ice sheet in the southeast. Not unexpectedly, along the periphery near the 1500 m snowline elevation, RCM densities are substantially greater (0.2–0.3 g/cm^3^) than those returned from the radar results (Fig. 2 and the elevation breakdowns included in the Supplementary Materials).

Ultimately, both model- and observation-based near-surface densities reveal similar long-term mean values, but each does appear to have their own idiosyncrasies. Variations in the former are driven almost exclusively by seasonal fluctuations within 500 m elevation of the snowline. HIRHAM5 and MAR surface densities across the higher-elevation interior of the GrIS are effectively homogeneous through time and space. When the radar-based densities do exhibit seasonal patterns, they often decrease in summer months. This is likely due to a change in the physical way radar signals are scattered from wet snow that is not being accounted for. In addition, the seasonally limited in situ database, hampers our ability to assess how the presence of melt changes the representative depths of the radar-derived densities. Outside of summer months though, when the GrIS surface can be considered frozen, CryoSat-2 and SARAL radar altimetry-based surface densities align with in situ measurements and reveal complex spatial pattern that are not currently reflected in state-of-the-art models. Extrapolating to mass balance calculations, these observation-based spatiotemporal patterns imply potential changes in both the magnitude and distribution of mass loss across the various sectors of the ice sheet. Ongoing work is actively investigating the quantitative impact of including observed densities in these calculations. Recognizing the slightly different representative depths of the Ku- and Ka-band results, the ability to investigate near-surface stratigraphy via dual-frequency altimetry is extremely relevant for future missions such as ESA CRISTAL. Now with quantified uncertainties and representative depths, radar altimetry-derived near-surface densities are a new and valuable dataset for integration in future climate studies.

## Online methods

### Derivation of near-surface densities from radar altimetry

For CryoSat-2, relevant surface echo powers are extracted from Baseline-E Level 1B (L1B) Low-Resolution Mode (LRM) data products in the GrIS interior and Full Bit Rate (FBR) (i.e., pre-SAR focusing) Synthetic Aperture Radar Interferometric (SARIn) data products nearer the margin. All SARAL surface echo powers are extracted from Sensor Geophysical Data Record (SGDR) data products. For each waveform, the surface echo power is defined as the maximum power measured in the 5% of the receive window following the position of the leading edge^23^. The leading edge is identified using a retracker based on the rate of change in waveform amplitude. Once extracted and corrected, CryoSat-2 SARIn echo powers are disregarded based on an internal data quality flag (CAL4). In the case of SARAL, an altimetric range over 1,000 km and/or a trailing edge variation flag are used to identify individual low-quality data points. Simultaneously large (> 0.10 degrees-squared pre-January 2019 and > 0.05 degrees-squared post-January 2019) average off-nadir angle is taken as indicative of a low-quality orbit. This latter consideration is in response to platform orientation concerns beginning with the failure of one onboard star tracker in early 2019^47^.

Surface echo powers are organized into monthly point clouds and, for a given location (e.g., in situ measurement, gridded pan-GrIS), the closest surface echo powers for the month in which the in situ measurement was collected are (1) extracted from each dataset (i.e., CryoSat-2 LRM, CryoSat-2 SARIn, and SARAL), (2) corrected for nadir surface slopes^23,48^ and (3) fed into the RSR analysis. As the initial study^23^ encountered difficulties recovering consistent CryoSat-2 surface roughness patterns when using 1,000 surface echo powers, in this study, CryoSat-2 SARIn coherent and incoherent powers are derived from the closest 12,000 samples. The 12,000 sample threshold is chosen as it is well beyond the range over which the comparison of coincident CryoSat-2 SARIn and SARAL surface roughness appears sensitive to the number of CryoSat-2 SARIn echoes (see Supplementary Materials). Drawing the 1,000 closest surface echo powers from the CryoSat-2 LRM and SARAL point clouds provided sufficient statistical representation. Due to the dominantly specular GrIS surface, CryoSat-2 SARIn density estimates are not strongly affected by the increase.

RSR processing involves fitting a homodyned K-distribution probability density function to the distribution of observed surface echo powers^20^. From the fit parameters of that distribution, the coherent and incoherent power components of the surface reflection can be derived and subsequently linked to surface conditions (i.e., density, roughness). Quality control criteria for the RSR results are a homodyned K-distribution fit correlation coefficient ≥ 0.96 and maximum search radii less than 40 km (SARAL), 25 km (CryoSat-2 SARIn), and 50 km (CryoSat-2 LRM). Any RSR results that do not meet these quality control criteria are not considered in subsequent comparisons against either in situ or model densities. The minimum correlation coefficient criterion is used to ensure the set of surface echo powers surrounding a specific location adhere to the theoretical expectation implicit in the RSR approach. Defining a maximum search radius is associated with spatial resolution and ensures the surface echo powers fed into the RSR analysis are not drawn from too large of an area surrounding a given position on the ice sheet.

To invert for the relative dielectric permittivity of the surface (that can subsequently be converted to a density) from the RSR outputs (i.e., coherent and incoherent powers), SARAL, CryoSat-2 SARIn and CryoSat-2 LRM calibration values must be defined. These values represent the relative strengths of SARAL or CryoSat-2 coherent powers for a known surface density. Fixing relative signal strengths to a known surface density allows for absolute changes in RSR coherent powers to then be tied to variations in the Fresnel reflection coefficient. Calibration values are determined using a subset of in situ measurements^29^ where the subsurface is considered homogeneous.

In keeping with the main analysis, only in situ measurements above the 1500 m elevation interval (the assumed snowline) are used in calibration. An in situ density measurement is considered homogeneous if the standard deviation in reported densities less than 0.05 g/cm^3^ over either the top 2 m (for SARAL) or top 3 m (CryoSat-2 LRM). The SARAL and CryoSat-2 LRM calibration powers along with the corresponding surface densities are 13.54 dB and 0.32 g/cm^3^ (SARAL) and 481 dB and 0.35 g/cm^3^ (CryoSat-2 LRM). There are not enough homogeneous in situ measurements that are both above 1500 m elevation and within the CryoSat-2 SARIn acquisition mask to facilitate a direct calibration directly. Instead, a relative calibration between contemporaneous (same month) and coincident (same location) CryoSat-2 LRM and CryoSat-2 SARIn coherent powers is used. These coincident locations occur along the border of the SARIn/LRM acquisition mask. The CryoSat-2 SARIn calibration values (i.e., relative surface echo power and surface density) are 570.7 dB and 0.35 g/cm^3^. This calibration procedure is the same that has been followed previously^23^ only now updated with the more comprehensive in situ database^29^ and Baseline-E CryoSat-2 data products.

Armed with the coherent and incoherent powers derived from the RSR analysis of CryoSat-2 and SARAL surface echoes and the corresponding calibration values, surface density is derived following the procedure used in the initial investigation^23^ now with two subtle modifications. The first is that in this study is that surface roughness is derived following a revised empirical approach^49^. The second is that a bootstrapping procedure is used to estimate uncertainties in the RSR outputs.

The bootstrapping uncertainty assessment takes place at 12 locations across the GrIS (6 covered by CryoSat-2 LRM data, 6 covered by CryoSat-2 SARIn data, all covered by SARAL data). At each location, the collection of closest surface echo powers (i.e., 1000 for CryoSat-2 LRM and SARAL, 12,000 for CryoSat-2 SARIn) are resampled 1000 times and the RSR results recalculated. The random sampling is done with replacement so some individual echo powers will appear multiple times in a randomly resampled bootstrap set. The standard deviation in the resulting distribution of the RSR outputs then provides a sense of the processing uncertainty inherent in the results. As with the conventional RSR analysis, only bootstrapped RSR outputs meeting the earlier established quality control criteria (i.e., minimum correlation coefficient and maximum search radius) are considered. That the standard deviation in the RSR results for a specific location and month be derived from at least 100 QC-passing bootstrap sets is also enforced. When applied to four roughly equally spaced, non-JJA (i.e., June, July, or August) months from the timeseries (i.e., October 2014, January 2018, December 2021, May 2023), the average standard deviation in the coherent power output from the RSR analysis is ±0.11 dB, ±0.05 dB, and ±0.36 dB for CryoSat-2 LRM, CryoSat-2 SARIn, and SARAL respectively.

The effective result of directly translating 2σ variations in coherent power to density is presented in the Supplementary Materials. As the coherent power is not linearly proportional to permittivity and density, the density variability due to the RSR processing is not fixed. Considering the range of recovered densities reported across the GrIS (~ 0.2–0.7 g cm^−3^; Figs. 2, 3 and 4, the variations associated with the RSR processing are generally smaller than (e.g., CryoSat-2 LRM and CryoSat-2 SARIn) or on par with (e.g., SARAL) the interquartile ranges derived from comparing the RSR density estimates to in situ measurements (Fig. 1).

### Resampling of depth-resolved in situ and model density profiles

The in situ density profiles are resampled to balance variability in the native in situ sampling intervals^29^ and how a potentially layered subsurface manifests in the reflected radar waveforms. In an ideal case, coherent echo powers could be compared against the effective reflectivity of a layered subsurface modelled using a transfer-matrix approach^21,50,51^. However, such a method requires in situ measurements that are sampled finely enough to capture every density variation as a function of depth. While neutron probe profiles are contained within the in situ database (sub-millimeter density sampling), there are not enough to facilitate a meaningful comparison. Simply applying a transfer matrix approach to the reported density profiles (e.g., from snow pits) would generate artificial reflections from averaged density contrasts and not represent the true strength or timing associated with what the radar altimeters signals encounter in the natural subsurface.

In addition, the ability to resolve reflections from every individual density contrast in the subsurface is limited by the bandwidth of the radar signals^24,28^ (320 MHz for CryoSat-2 and 500 MHz for SARAL). CryoSat-2 and SARAL sample the reflected waveforms at their respective free-space range resolution, such that reflections from smaller subsurface layers cannot be reliably resolved. As such, to be comparable to each satellite dataset, in situ density profiles are resampled three times, once for each of the free-space range window lengths used to sample the reflected radar altimetry waveforms (i.e., 0.4684 m for CryoSat-2 LRM, 0.2342 m for CryoSat-2 SARIn, and 0.3000 m for SARAL).

The final in situ density profiles represent the average, thickness-weighted measured densities within each resampled depth increment. Where an in situ density profile does not entirely extend through an entire depth increment (e.g., a shallow measurement of surface snow density), the remaining portion of the depth increment is treated as homogeneous but the resampled profile extends no further. Scaling the free-space range windows based on an arbitrary subsurface radar wave velocity does not affect the results.

At their respective positional and temporal sampling, both MAR^30,31^ and HIRHAM5^11,32,33^ return modelled density estimates with depth in the GrIS near-surface. For MAR, the subsurface scheme is based on the CROCUS model^52,53^. The subsurface is partitioned into 18 layers of varying thicknesses and densities determined by location specific compaction rates (due to modelled snow deposition) as well as liquid water retention and refreezing processes. For HIRHAM5, the subsurface is separated into 32 layers of varying thicknesses. Mass is added to the top of the HIRHAM5 subsurface model via snow, rainfall, condensation and deposition processes and advected downward, while processes that remove mass (e.g., evaporation, runoff, and sublimation) cause ice to be advected upward into the bottom-most layer. The transfer of other properties (e.g., snow/ice/water fractions, sensible heat, density and grain size) between vertical layers is also considered. For comparison with the radar altimetry results, model density profiles are resampled from their native variable depth intervals to a constant depth interval using the same bandwidth-based interval thicknesses applied to the in situ data.

### Co-locating observation- and model-based densities

In their native form, the observation- and model-based densities compared in this study are not at the same location nor do they have the same spatial resolution. Observation, HIRHAM5 and MAR densities are reported across independent square grids with individual node spacings of 5 km, 5 km, and 10 km respectively. The reported spatial resolutions for HIRHAM5 and MAR are 5.5 km^32^ and 20 km^31^ respectively. Spatial resolution of the densities derived from SARAL and CryoSat-2 surface echo powers is not constant. How wide of an area the requisite number of surface echo powers needed to perform the RSR processing is drawn from depends on the satellite coverage of that region at that time. However, the radius of that area is not allowed to increase indefinitely as, as introduced earlier in these Online Methods, maximum search radii are used as quality control metrics when assessing the reliability of the RSR output.

To get a sense of the inherent spatial variability in the different density datasets, this study makes use of empirical variograms. Example monthly variograms for both 2015 and 2021 are presented in the Supplementary Materials. What these variograms present are the semivariances in the reported densities (at their individual native posting intervals) as a function of the distance between pairs of points being compared (i.e., the lag). A greater semivariance at a specific lag is indicative of more spatial variability in that dataset. We find that spatial variability in the radar-derived densities is roughly comparable to those in the models (e.g., semivariances are generally of similar magnitude) but there are exceptions. For example, consider densities derived from CryoSat-2 LRM surface echoes (i.e., those in the high-elevation interior of the ice sheet) with those extracted from HIRHAM5 and MAR for a similar depth interval. In both 2015 and 2021, except for HIRHAM5 at very short lags, the CryoSat-2 LRM density semivariances tend to always be greater than those from the models indicating increased spatial homogeneity. In terms of how spatial variability varies across the different density datasets with time, the empirical variograms reveal a pattern similar to what was observed in Figs. 2 and 3, radar-observed densities vary more through time in the interior of the ice sheet while model-based densities vary more in time and space at lower elevations.

Ultimately, because of the general agreement in the degree of spatial variability across the different density datasets, the decision was made to co-locate results at nodes of the 10-by-10 km MAR grid. This also represents the coarsest native spatial spacing of any of the density datasets. Using the MAR grid means no dataset is resampled to a spatial interval smaller than its native grid; the HIRHAM5 and radar-based densities are smoothed rather than the MAR densities interpolated. The comparative HIRHAM5 and radar observation-based densities defined at the locations of the MAR estimates are the mean values of those datasets for results found within 10 km of each MAR grid node.

## Electronic supplementary material

Below is the link to the electronic supplementary material.

Supplementary Material 1

## Data Availability

Quality-controlled RSR results at the locations of the in situ density measurements used in calibration/validation as well as the monthly pan-GrIS results are available through http://data.dtu.dk/ with https://doi.org/10.11583/DTU.27612045.v1.
