# Supplementary material for: Novel insight into the spatiotemporal distribution of Greenland ice sheet surface densities from eleven years of satellite radar altimetry
Source: Sci Rep. 2025 May 17;15:17156. doi: 10.1038/s41598-025-02403-2 (PMC12085595; doi:10.1038/s41598-025-02403-2)
Supplement: Supplementary file 1 — Supplementary Material 1 [file 41598_2025_2403_MOESM1_ESM.docx]

**Supplemental Information for “Novel insight into the spatiotemporal distribution of Greenland ice sheet surface densities from eleven years of satellite radar altimetry”**

Kirk M. Scanlan^1^, Anja Rutishauser^2^, Nicolaj Hansen^1,3^, and Sebastian B. Simonsen^1^

^1^DTU Space, Technical University of Denmark, 2800 Kgs. Lyngby, Denmark

^2^Geological Survey of Denmark and Greenland, 1350 Copenhagen, Denmark

^3^Department of National Centre for Climate Research (NCKF), Danish Meteorological Institute, 2100 Copenhagen, Denmark

*Incremental Results*

This Supplemental Information section contains incremental results in support of the figures presented in the main body of the manuscript as well as the Online Methods. Specifically, Figures S1-S10 are provided in support of Figure 1 and present the comparison of SARAL and CryoSat-2 densities against in situ measurements for each of the ten different permittivity-to-density conversion models considered as part of this work. Figures S11-S13 are provided in support of Figure 2 and present the radar- and RCM-based density timeseries as a function of surface elevation (SARAL in Figure S11, CryoSat-2 SARIn in Figure S12, and CryoSat-2 LRM in Figure S13). Figure S14 is provided in support of Figures 4 and 5 and presents maps of the number of individual months between May 2013 and December 2023 that return valid SARAL and CryoSat-2 (SARIn and LRM) RSR results. Figure S15 is provided in support of the Online Methods and demonstrates the sensitivity of surface roughness derived from CryoSat-2 SARIn data products to the number of surface echo powers incorporated in the RSR processing. Figure S16 is provided in support of the Online Methods to demonstrate how uncertainties in the coherent power component, determined from random resampling of the surface echo powers input into the RSR processor (i.e., bootstrapping), would translate to density estimates. Finally Figures S17-S22 are provided in support of the Online Methods and contain empirical semivariograms derived from monthly radar- and RCM-based densities for both 2015 (Figures S16-S18) and 2021 (Figures S19-S21).


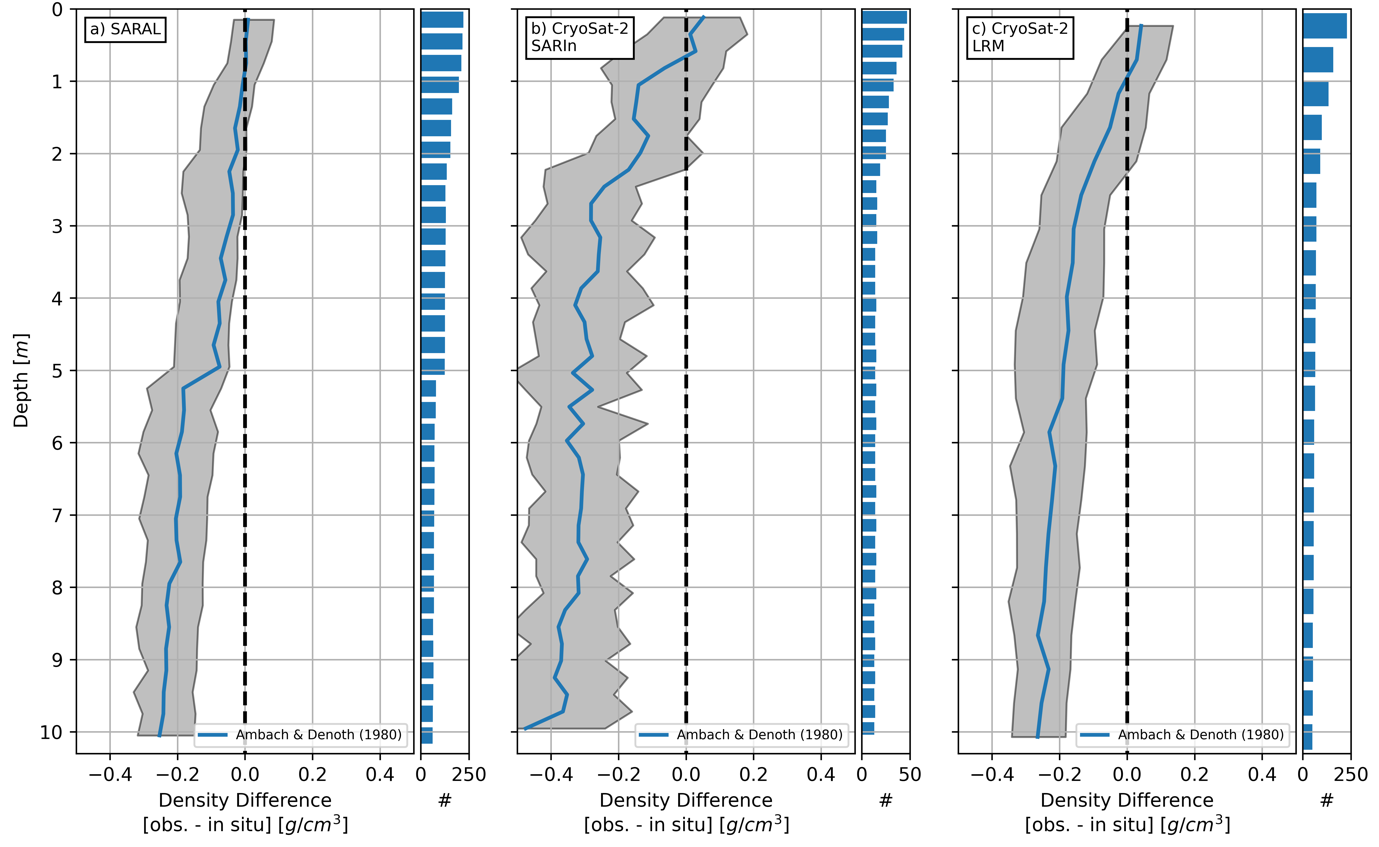


**Figure S1.** Comparison of a) SARAL, b) CryoSat-2 SARIn, and c) CryoSat-2 LRM densities derived using the Ambach and Denoth^34^ permittivity-to-density conversion model against in situ measurements. Shaded areas denote the interquartile range. Radar-specific representative depths are defined where the median density difference is zero. Bar plots represent the number of radar-to-model density comparisons within each depth increment.


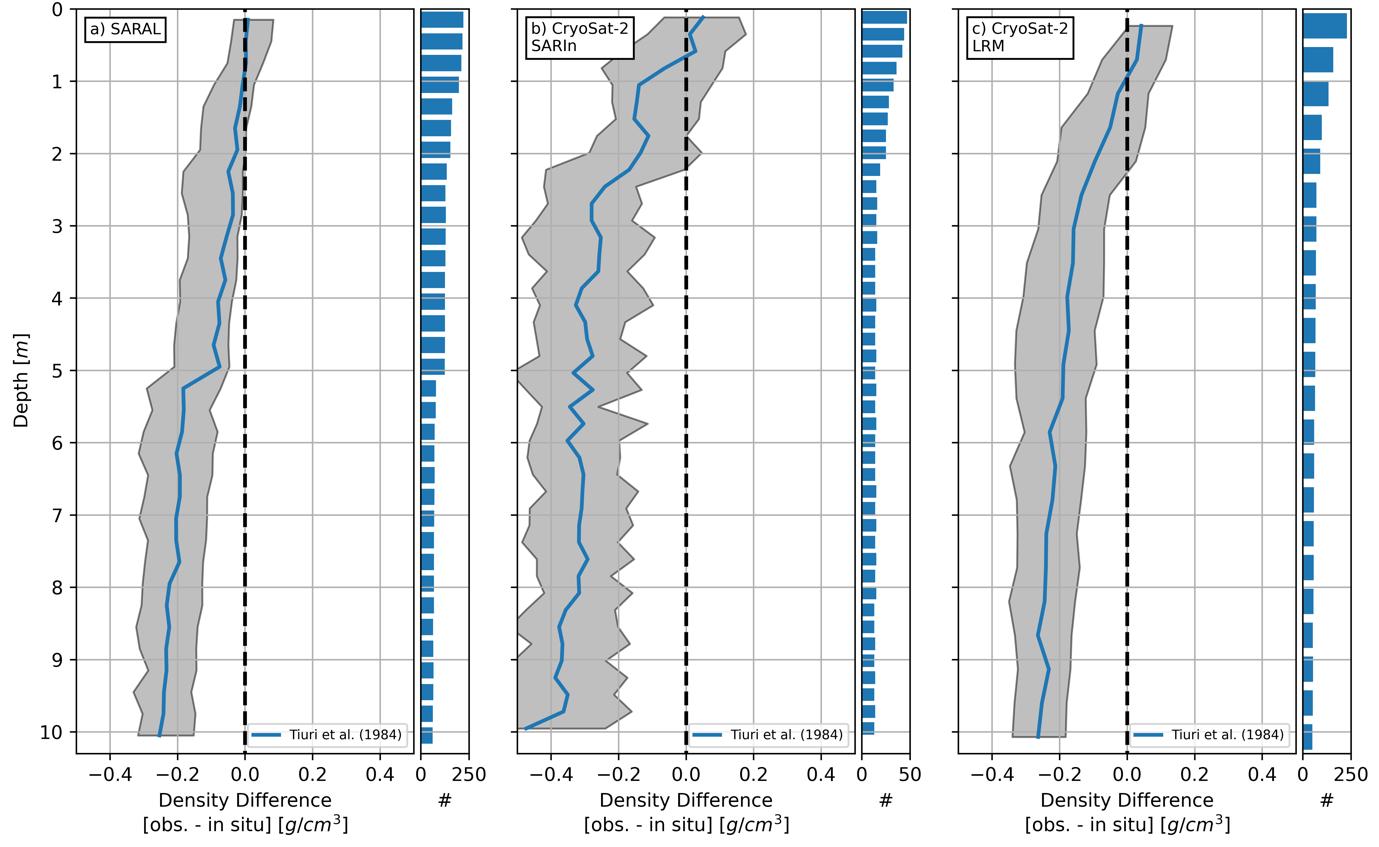


**Figure S2.** Comparison of a) SARAL, b) CryoSat-2 SARIn, and c) CryoSat-2 LRM densities derived using the Tiuri et al.^35^ permittivity-to-density conversion model against in situ measurements. Shaded areas denote the interquartile range. Radar-specific representative depths are defined where the median density difference is zero. Bar plots represent the number of radar-to-model density comparisons within each depth increment.

**
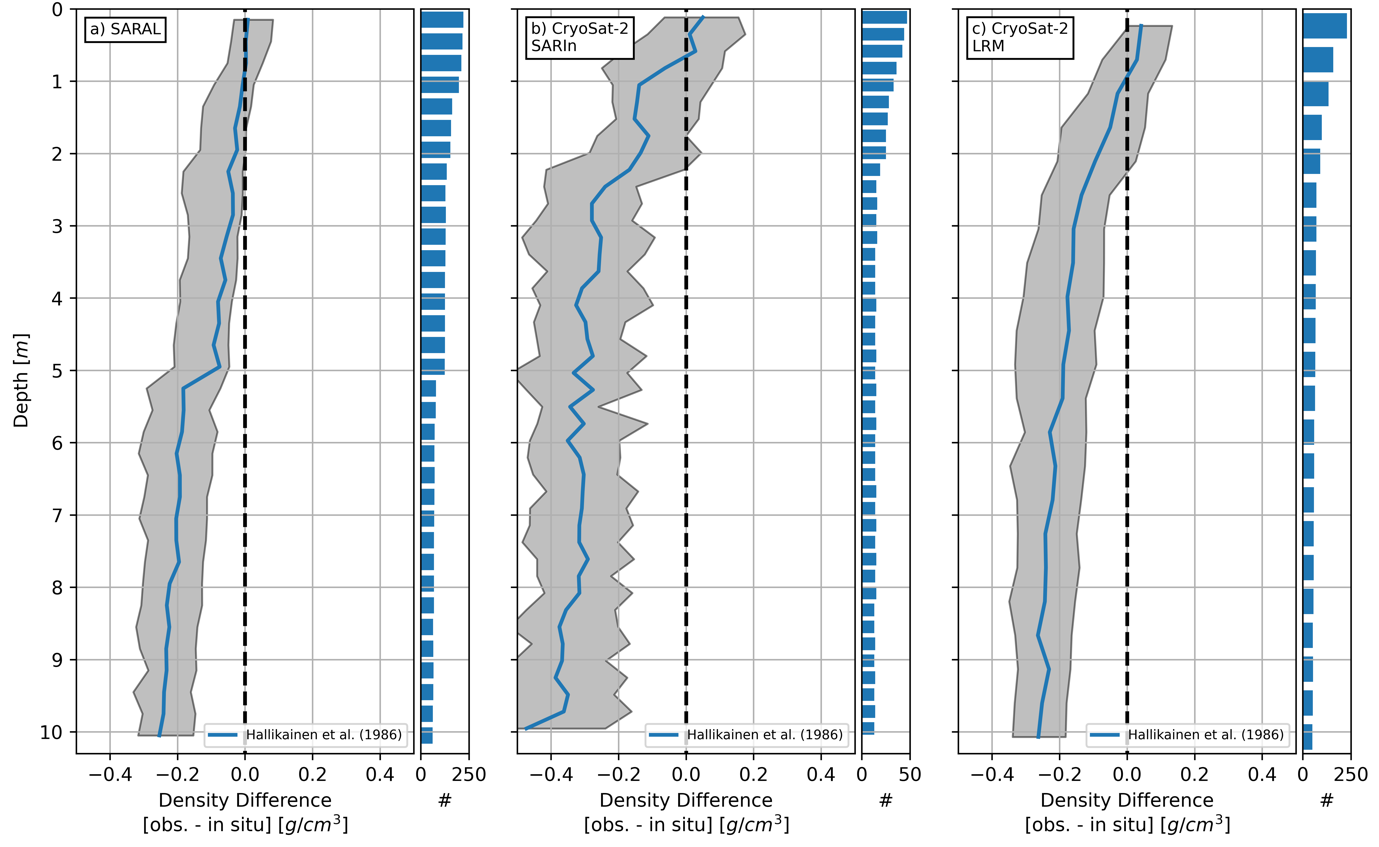
**

**Figure S3.** Comparison of a) SARAL, b) CryoSat-2 SARIn, and c) CryoSat-2 LRM densities derived using the Hallikainen et al.^36^ permittivity-to-density conversion model against in situ measurements. Shaded areas denote the interquartile range. Radar-specific representative depths are defined where the median density difference is zero. Bar plots represent the number of radar-to-model density comparisons within each depth increment.

**
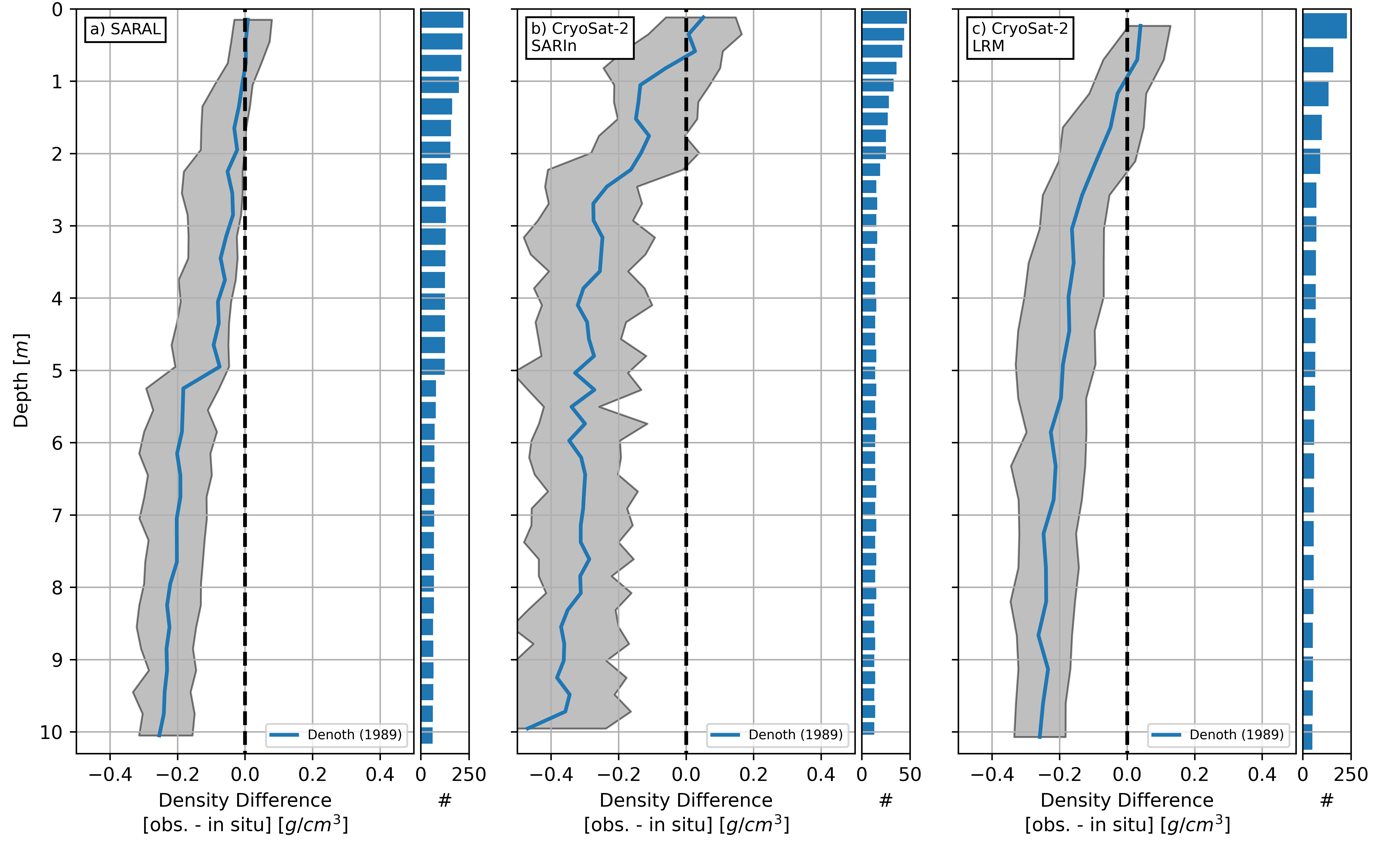
**

**Figure S4.** Comparison of a) SARAL, b) CryoSat-2 SARIn, and c) CryoSat-2 LRM densities derived using the Denoth^37^ permittivity-to-density conversion model against in situ measurements. Shaded areas denote the interquartile range. Radar-specific representative depths are defined where the median density difference is zero. Bar plots represent the number of radar-to-model density comparisons within each depth increment.


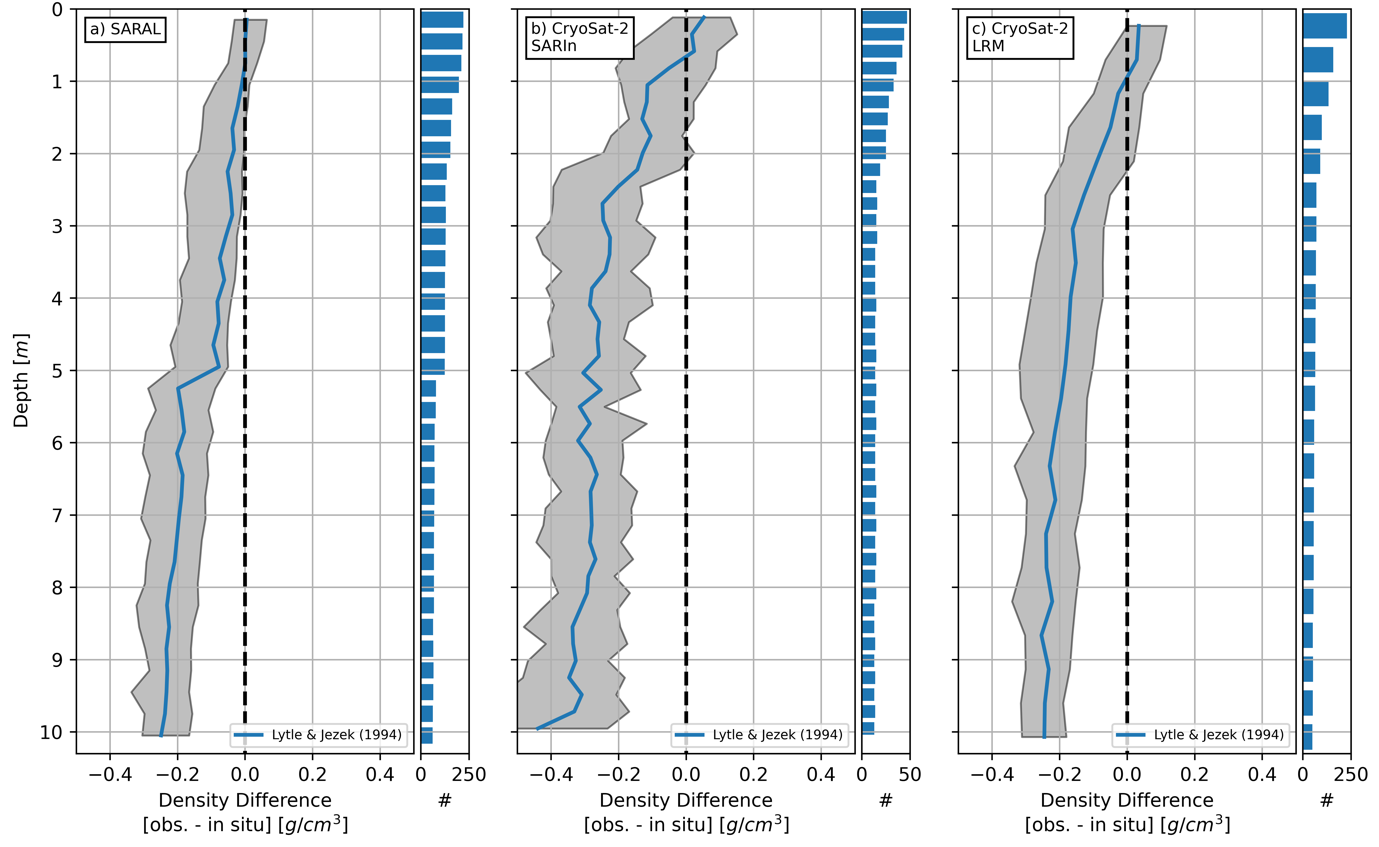


**Figure S5.** Comparison of a) SARAL, b) CryoSat-2 SARIn, and c) CryoSat-2 LRM densities derived using the Lytle and Jezek^38^ permittivity-to-density conversion model against in situ measurements. Shaded areas denote the interquartile range. Radar-specific representative depths are defined where the median density difference is zero. Bar plots represent the number of radar-to-model density comparisons within each depth increment.


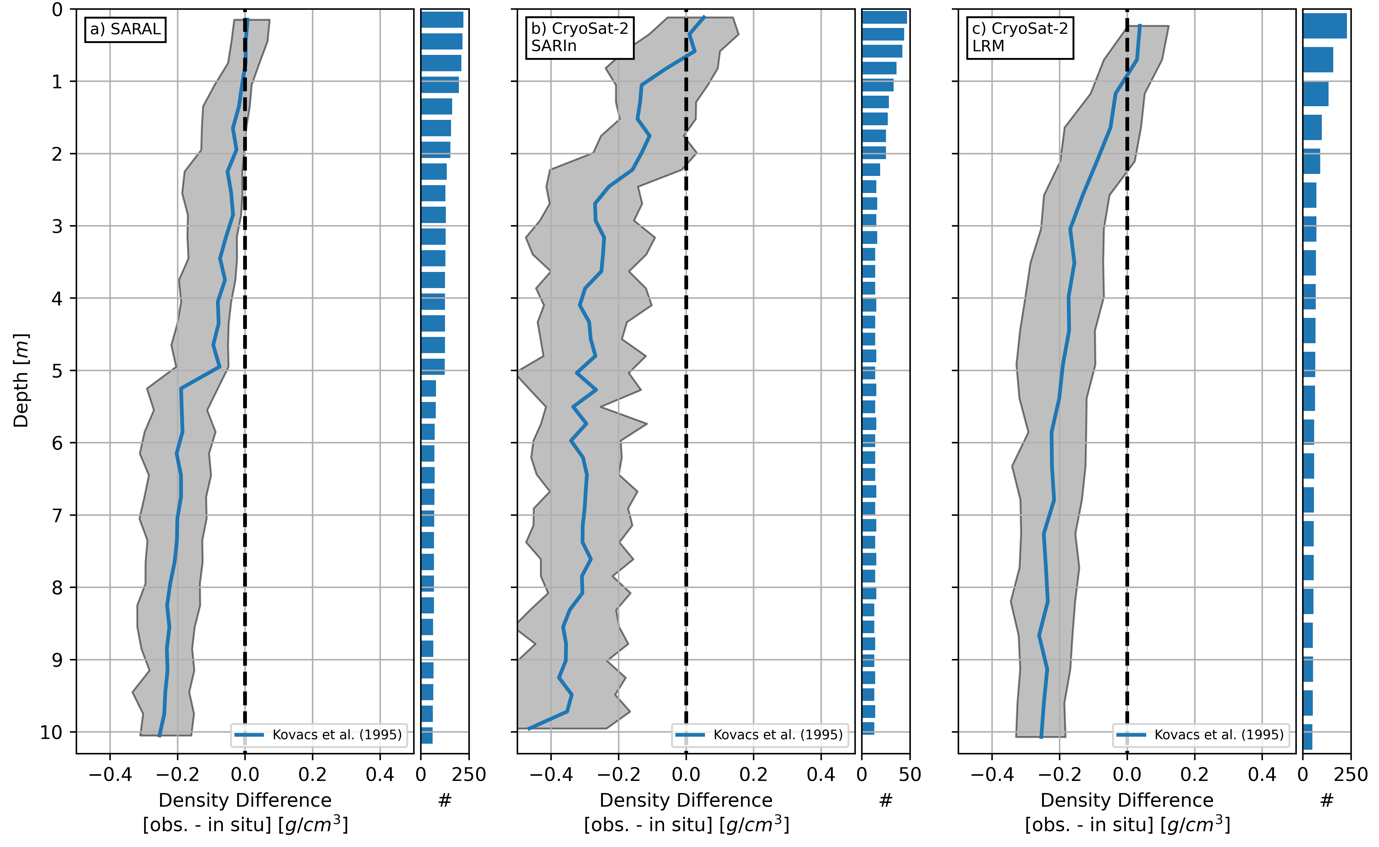


**Figure S6.** Comparison of a) SARAL, b) CryoSat-2 SARIn, and c) CryoSat-2 LRM densities derived using the Kovacs et al.^39^ permittivity-to-density conversion model against in situ measurements. Shaded areas denote the interquartile range. Radar-specific representative depths are defined where the median density difference is zero. Bar plots represent the number of radar-to-model density comparisons within each depth increment.


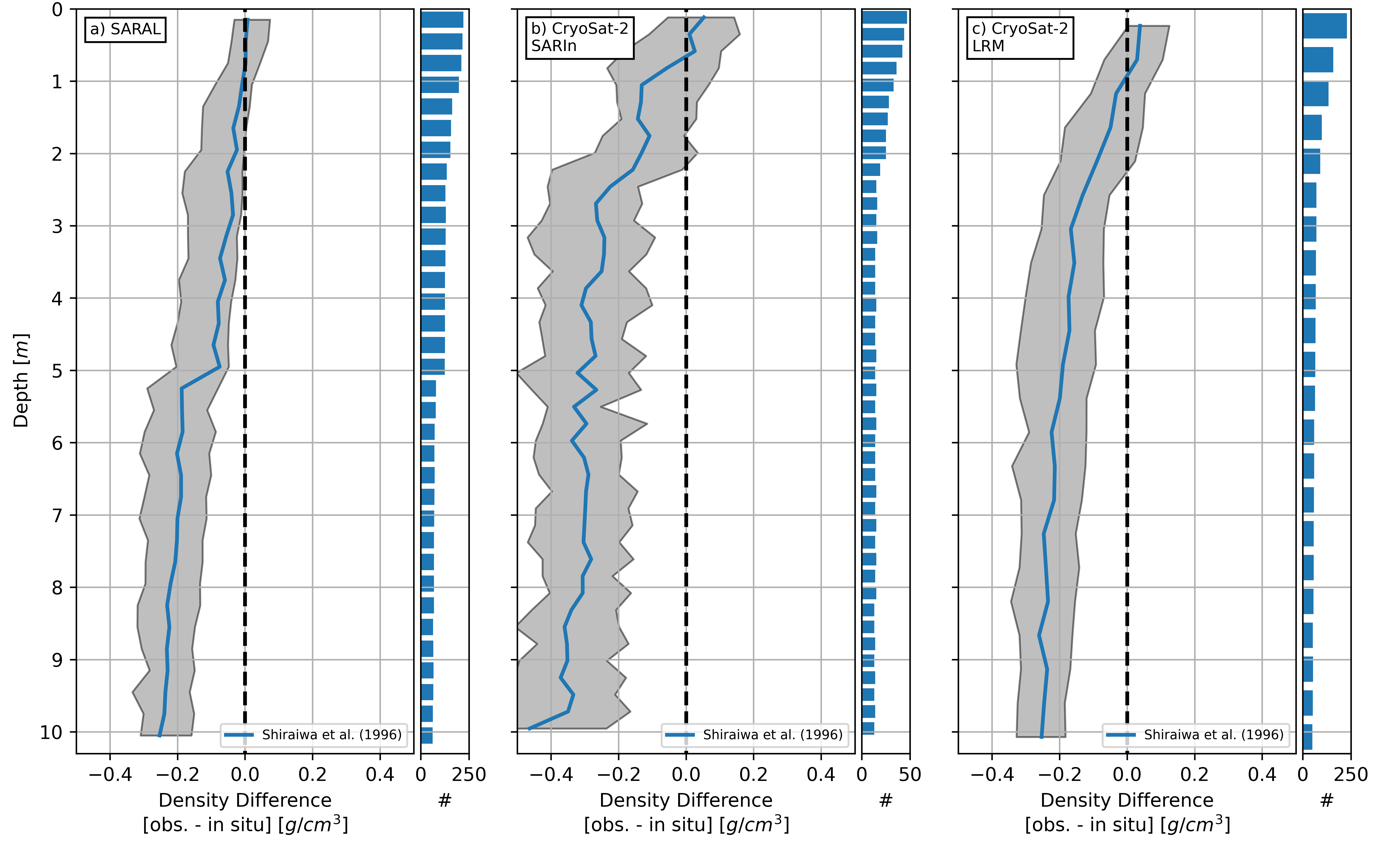


**Figure S7.** Comparison of a) SARAL, b) CryoSat-2 SARIn, and c) CryoSat-2 LRM densities derived using the Shiraiwa et al.^40^ permittivity-to-density conversion model against in situ measurements. Shaded areas denote the interquartile range. Radar-specific representative depths are defined where the median density difference is zero. Bar plots represent the number of radar-to-model density comparisons within each depth increment.


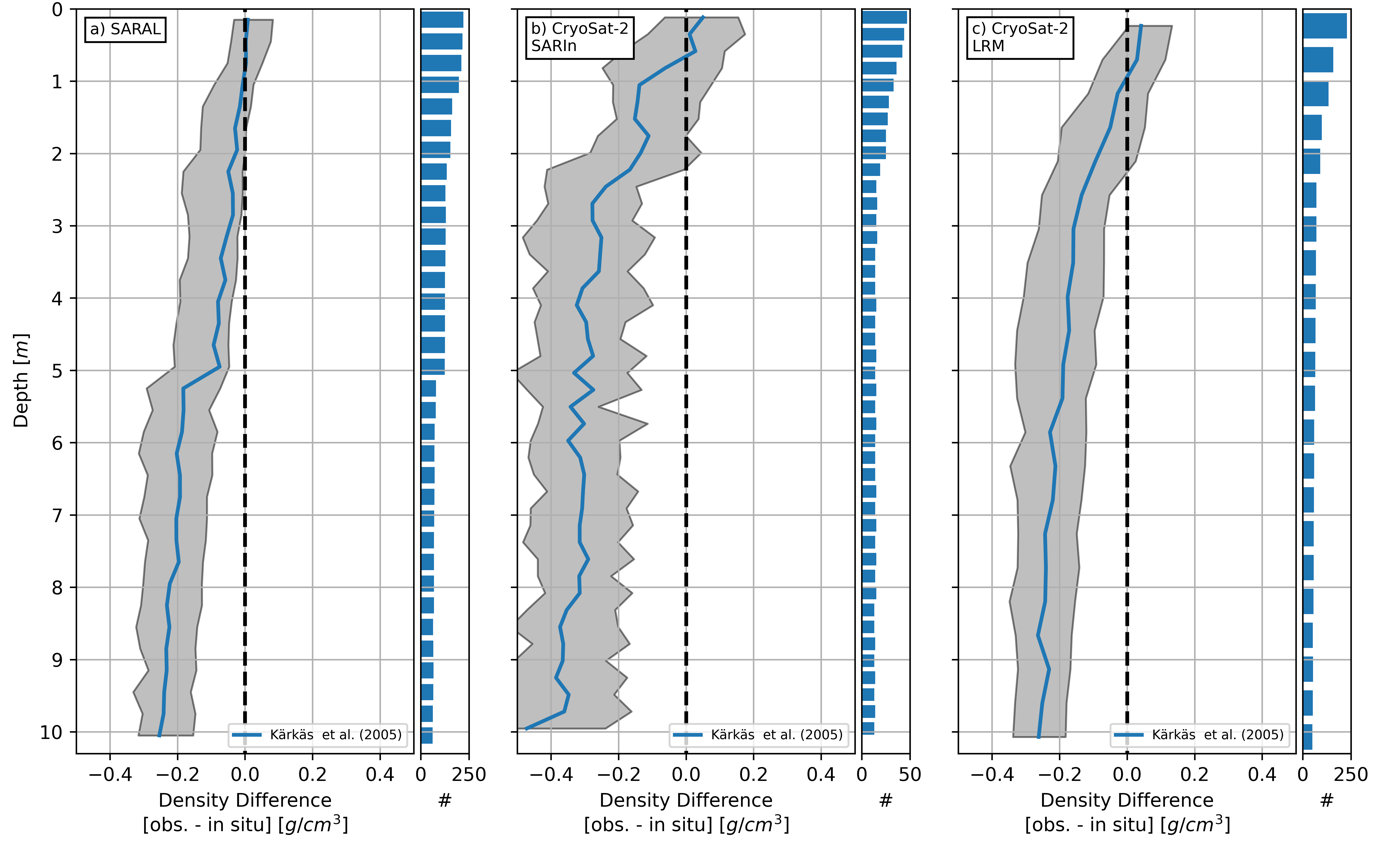


**Figure S8.** Comparison of a) SARAL, b) CryoSat-2 SARIn, and c) CryoSat-2 LRM densities derived using the Kärkäs et al.^41^ permittivity-to-density conversion model against in situ measurements. Shaded areas denote the interquartile range. Radar-specific representative depths are defined where the median density difference is zero. Bar plots represent the number of radar-to-model density comparisons within each depth increment.


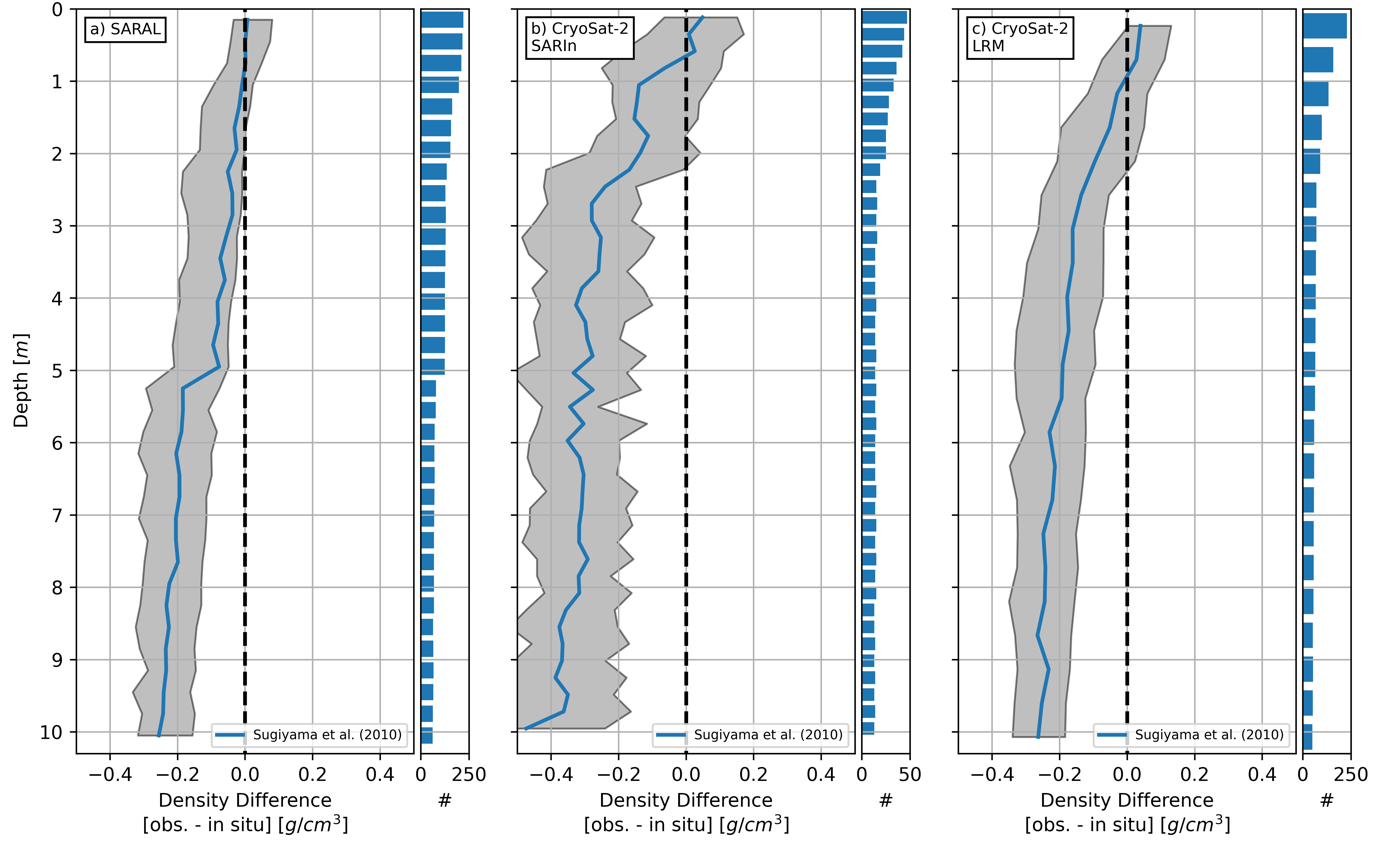


**Figure S9.** Comparison of a) SARAL, b) CryoSat-2 SARIn, and c) CryoSat-2 LRM densities derived using the Sugiyama et al.^42^ permittivity-to-density conversion model against in situ measurements. Shaded areas denote the interquartile range. Radar-specific representative depths are defined where the median density difference is zero. Bar plots represent the number of radar-to-model density comparisons within each depth increment.


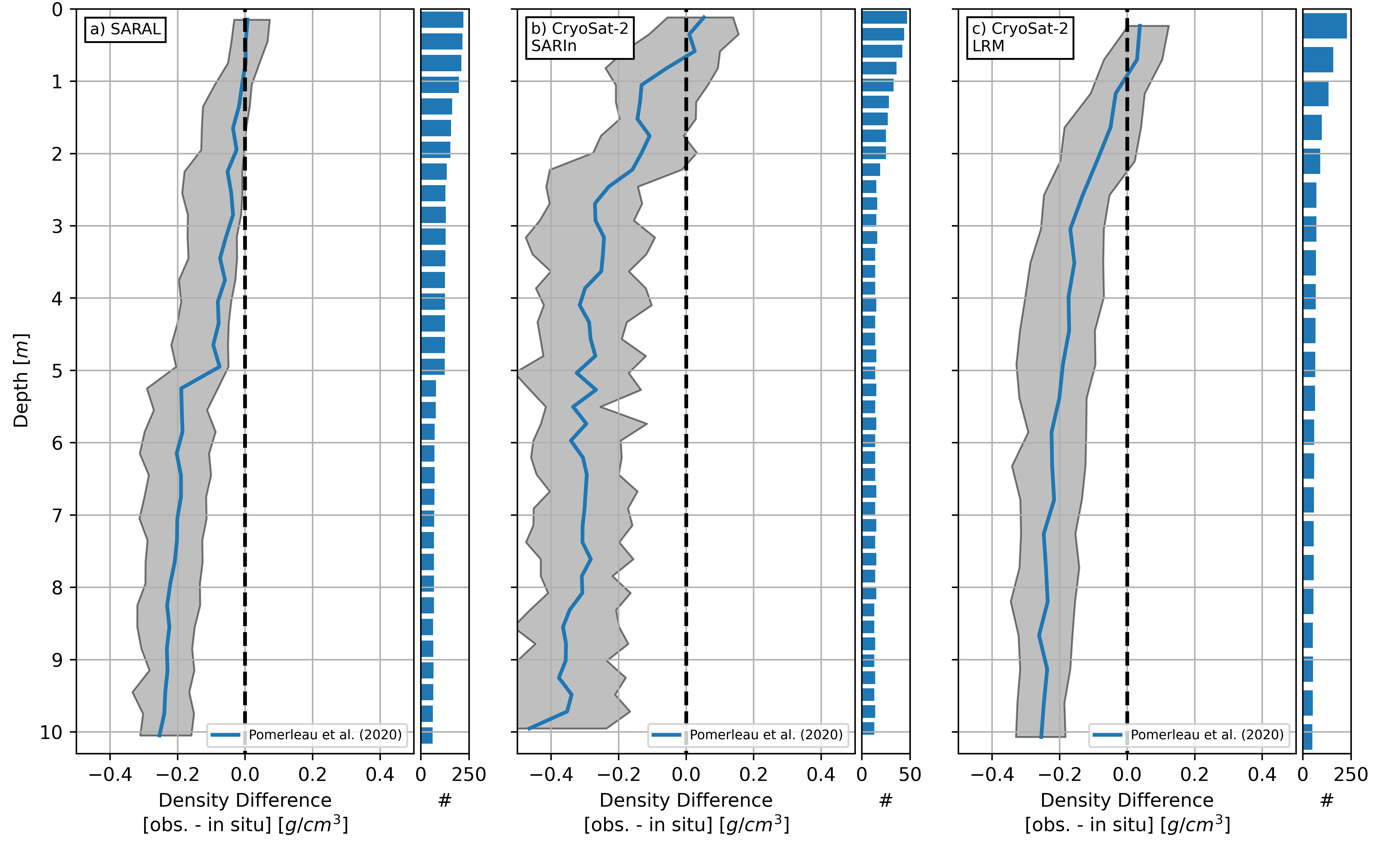


**Figure S10.** Comparison of a) SARAL, b) CryoSat-2 SARIn, and c) CryoSat-2 LRM densities derived using the Pomerleau et al.^43^ permittivity-to-density conversion model against in situ measurements. Shaded areas denote the interquartile range. Radar-specific representative depths are defined where the median density difference is zero. Bar plots represent the number of radar-to-model density comparisons within each depth increment.

**
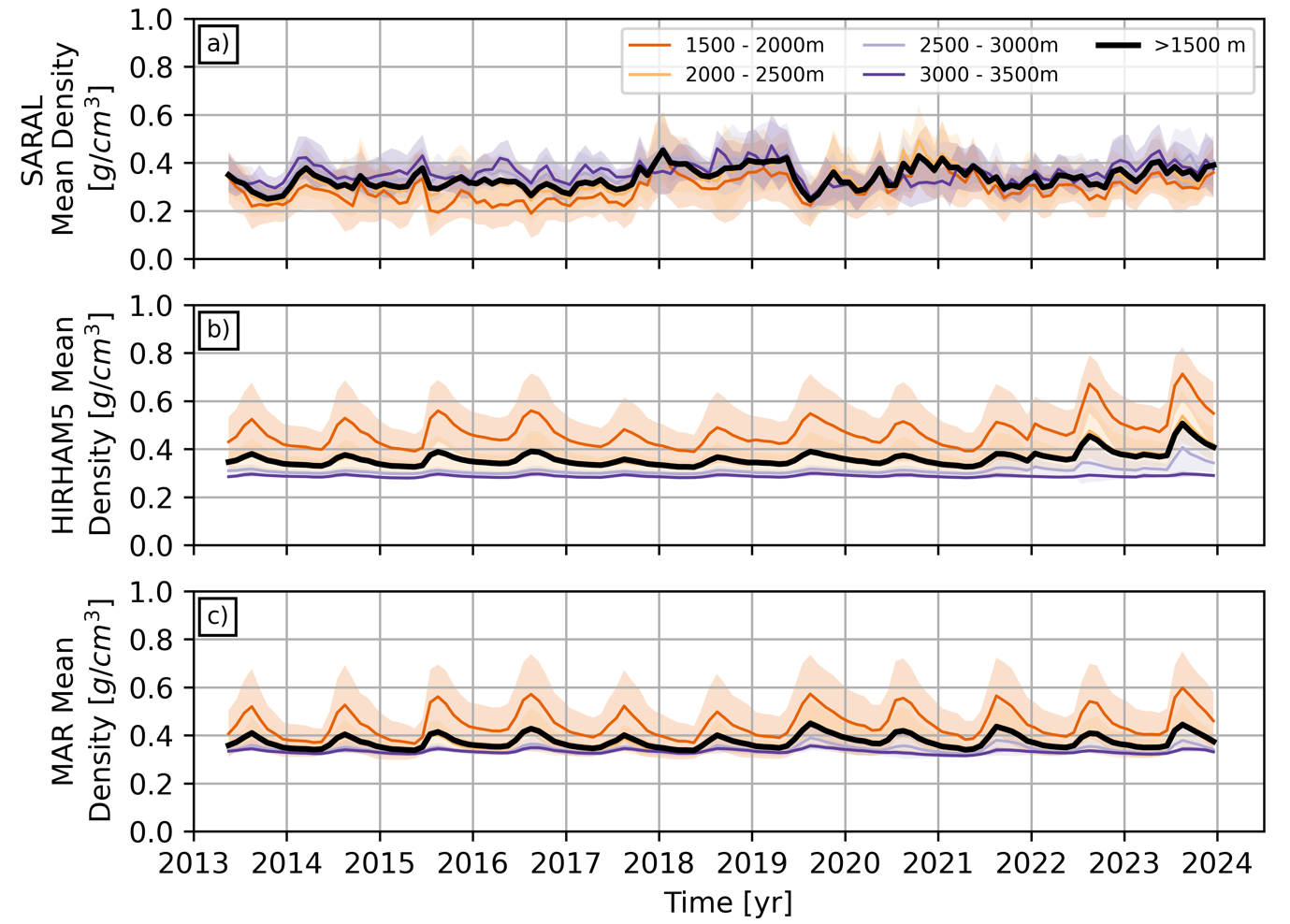
**

**Figure S11.** Long-term SARAL and RCM mean GrIS density timeseries for the SARAL representative depth interval (0-1.0 m) broken down by 500 m elevation interval along the with >1500 m (the same as is shown in Figure 2). Shaded area around the mean timeseries represents one standard deviation in reported densities for that month. Variability in RCM density is driven predominantly by the lowest surface elevations.

**
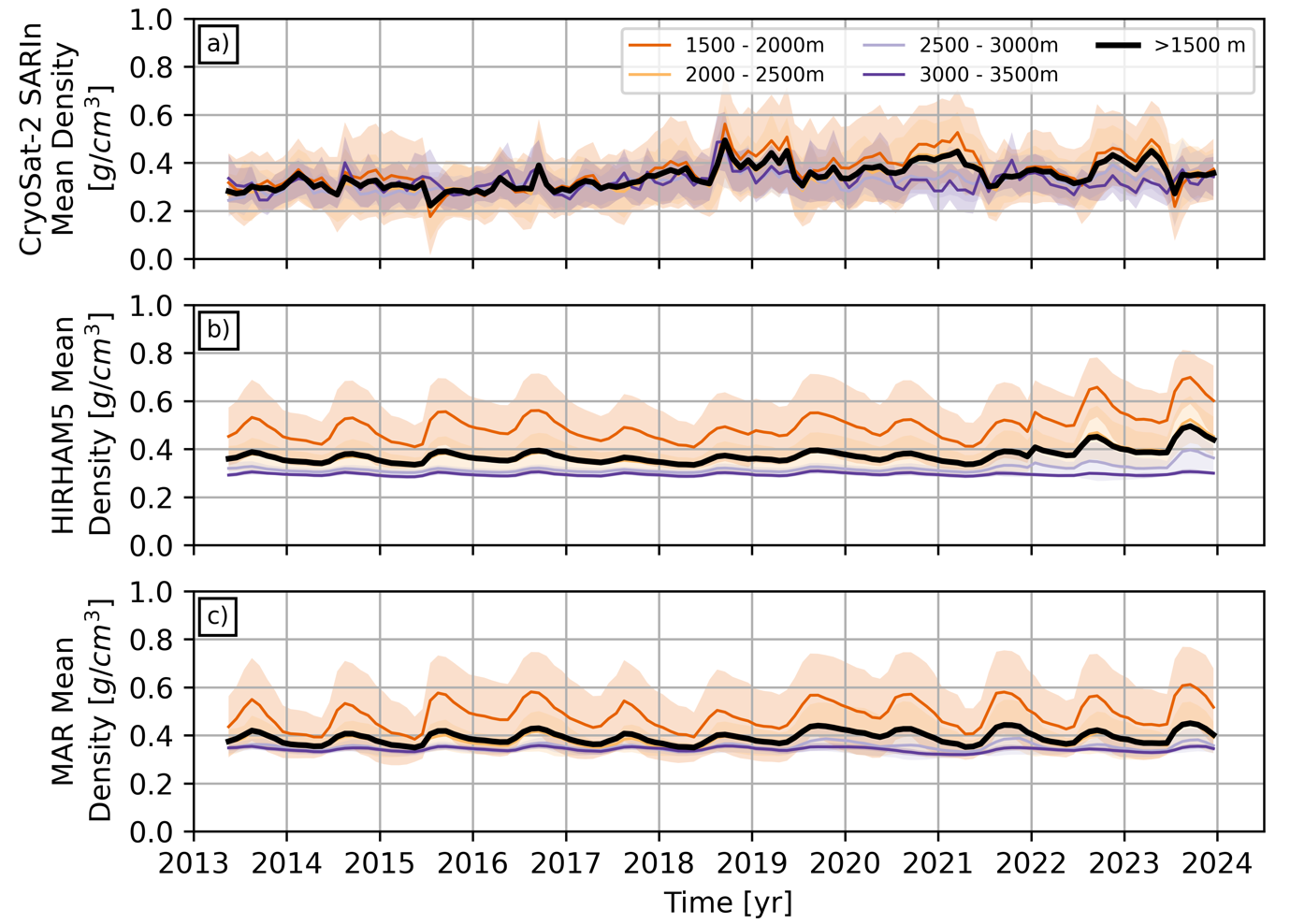
**

**Figure S12.** Long-term CryoSat-2 SARIn and RCM mean GrIS density timeseries for the CryoSat-2 SARIn representative depth interval (0.5-0.8 m) broken down by 500 m elevation interval along the with >1500 m (the same as is shown in Figure 2). Shaded area around the mean timeseries represents one standard deviation in reported densities for that month. Variability in RCM density is driven predominantly by the lowest surface elevations.

**
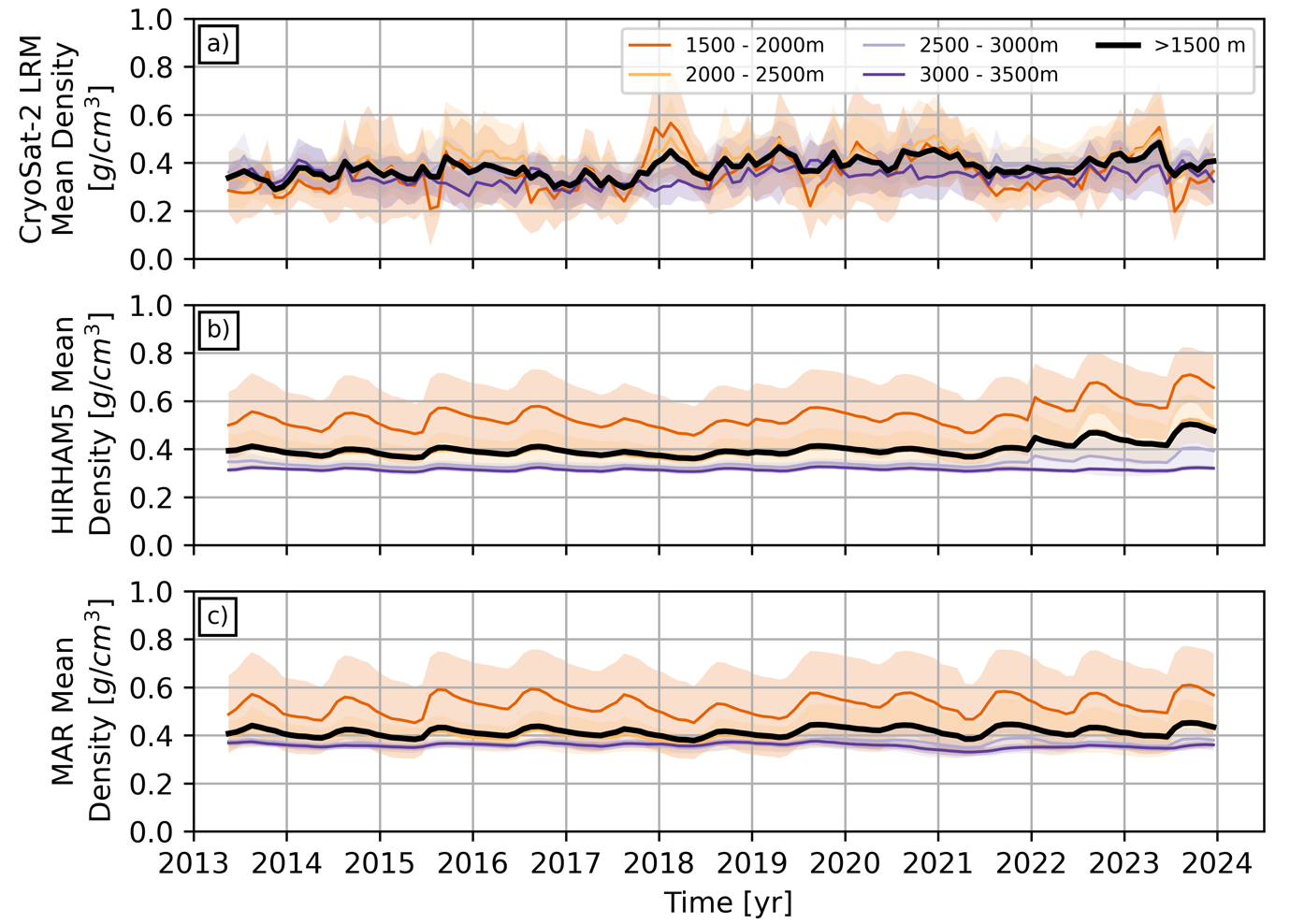
**

**Figure S13.** Long-term CryoSat-2 LRM and RCM mean GrIS density timeseries for the CryoSat-2 LRM representative depth interval (0.75-1.25 m) broken down by 500 m elevation interval along the with >1500 m (the same as is shown in Figure 2). Shaded area around the mean timeseries represents one standard deviation in reported densities for that month. Variability in RCM density is driven predominantly by the lowest surface elevations.


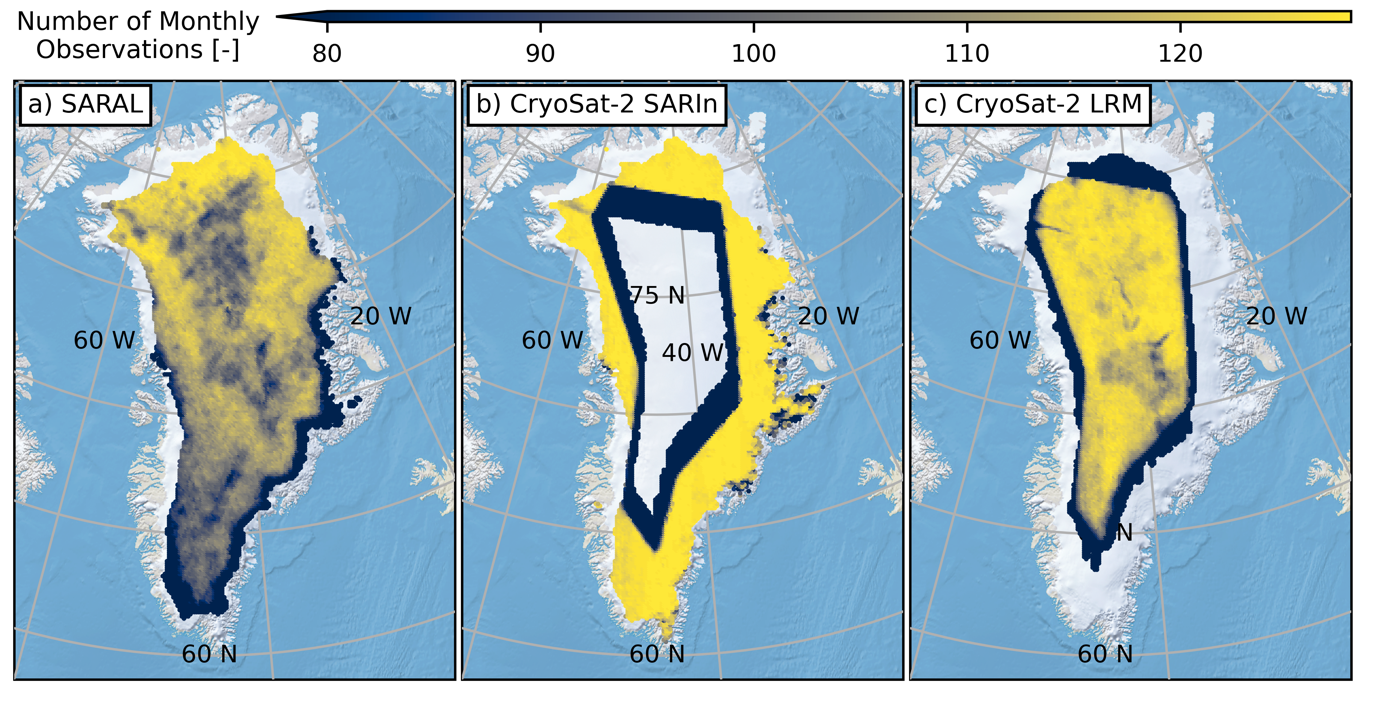


**Figure S14.** Maps of the number of months returning valid a) SARAL, b) CryoSat-2 SARIn, and c) CryoSat-2 LRM RSR results across the 128-month May 2013 through December 2023 time period. This figure is made in Python3 (v3.8.8) using the public domain Natural Earth II basemap.

**
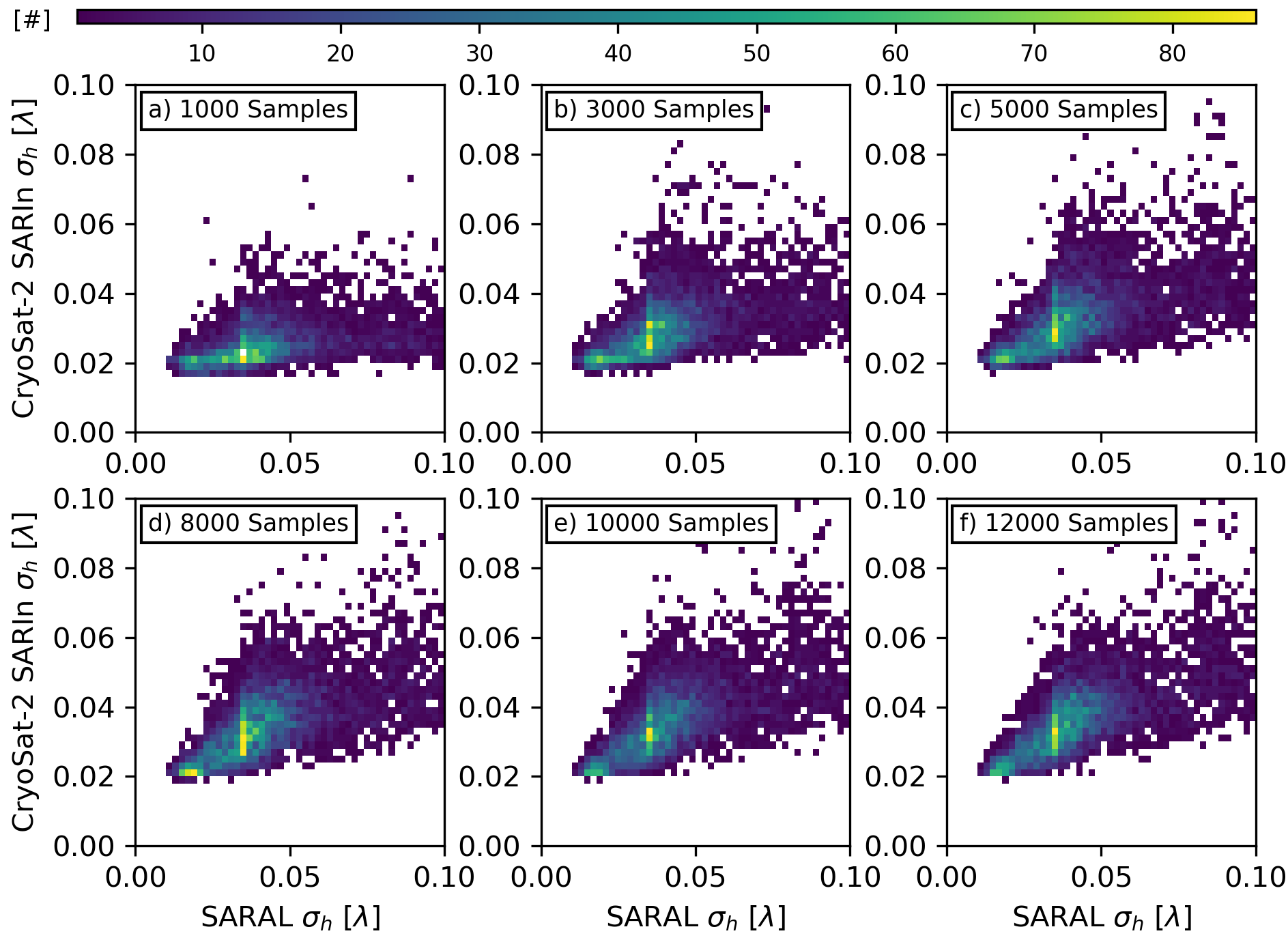
**

**Figure S15.** Comparison of coincident May 2015 RSR-derived RMS heights derived from 1,000 SARAL surface echoes and a variable number of CryoSat-2 SARIn surface echoes. By 12,000 CryoSat-2 SARIn surface echoes [f)], the distribution stabilizes and the CryoSat-2 SARIn RSR results are assumed to be no longer sensitive to the number of surface echoes.

**
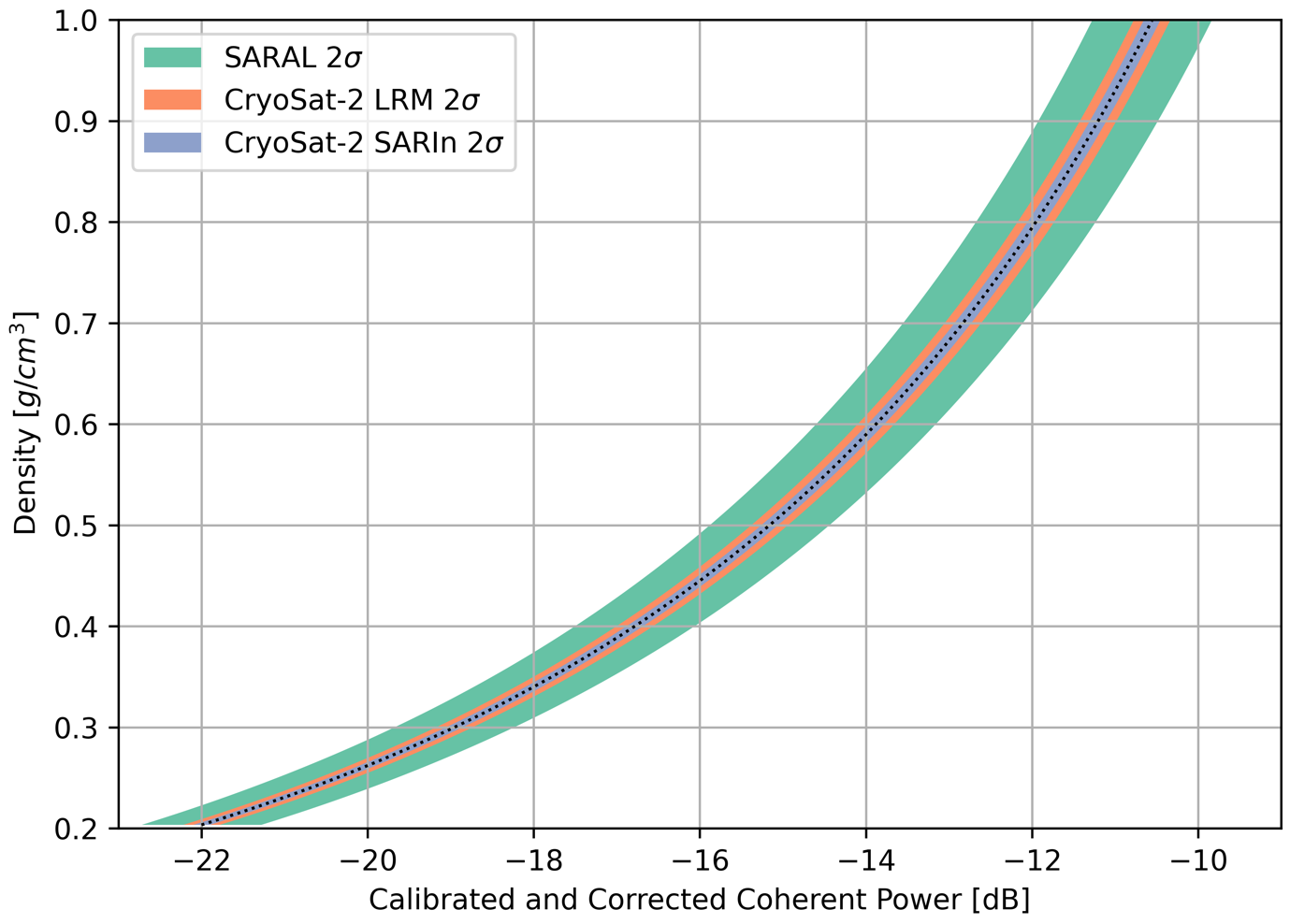
**

**Figure S16.** Influence of mean 2σ RSR processing uncertainties as determined by bootstrapping on associated density estimates. Densities are derived using following the Pomerleau et al.^43^ permittivity-to-density conversion model. The RSR algorithm likely produces more consistent coherent power estimates for the CryoSat-2 SARIn data compared to the CryoSat-2 LRM or SARAL due to the greater spatial density of surface echo powers.

**
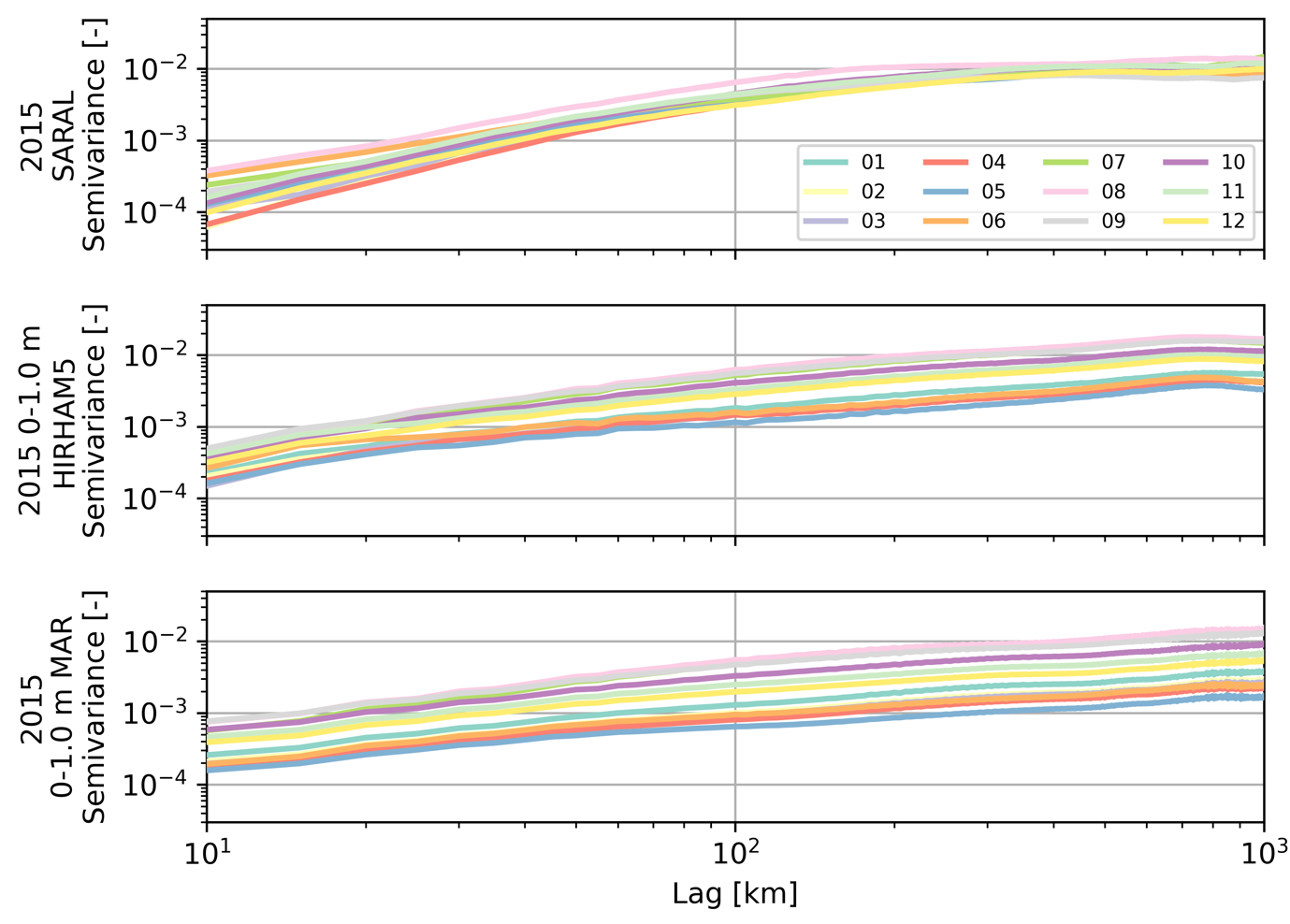
**

**Figure S17.** 2015 monthly empirical semivariograms derived from a) SARAL densities as well as b) HIRHAM5 and c) MAR results within the SARAL representative depth interval (0-1.0 m).


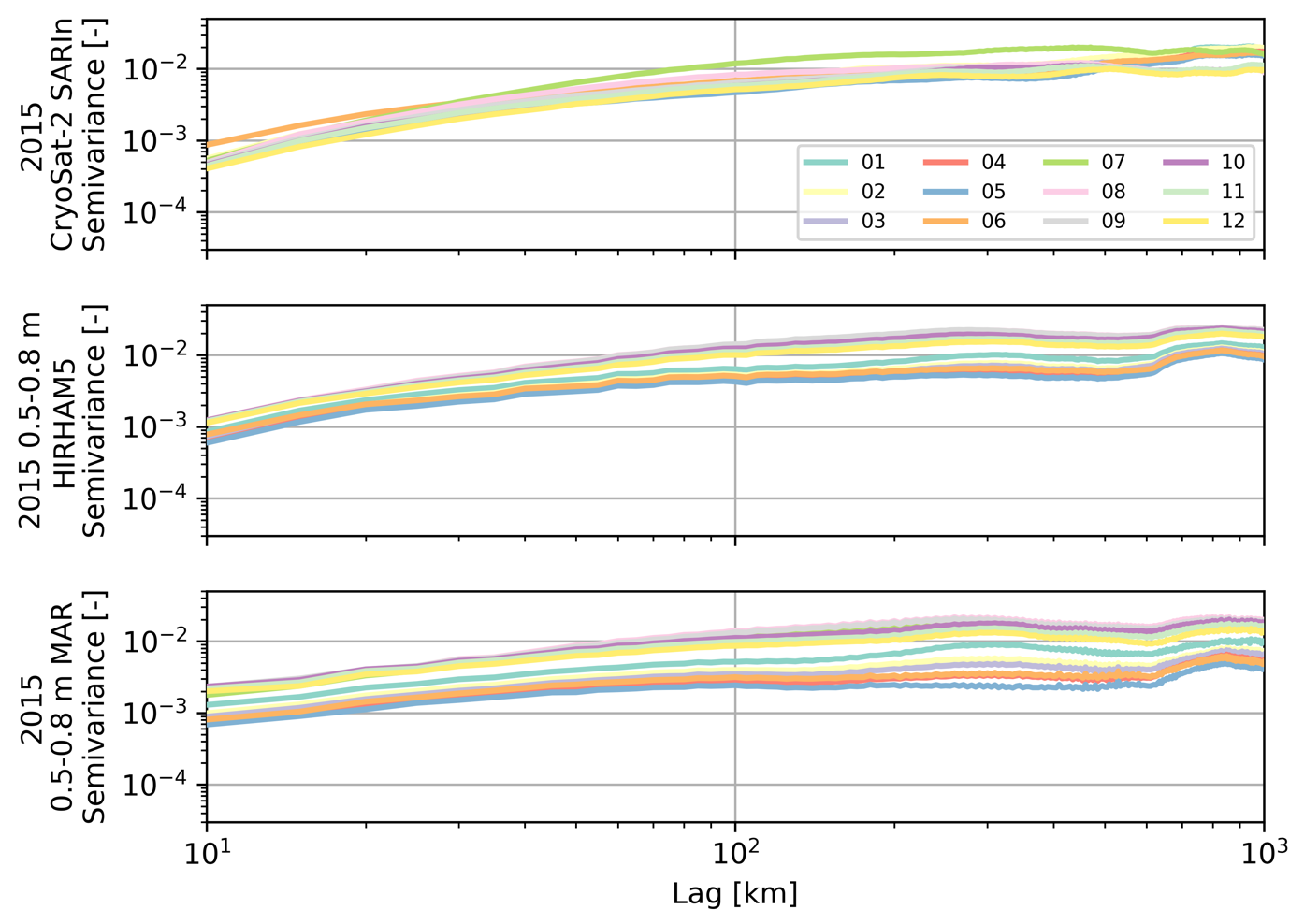


**Figure S18.** 2015 monthly empirical semivariograms derived from a) CryoSat-2 SARIn densities as well as b) HIRHAM5 and c) MAR results within the CryoSat-2 SARIn representative depth interval (0.5-0.8 m).


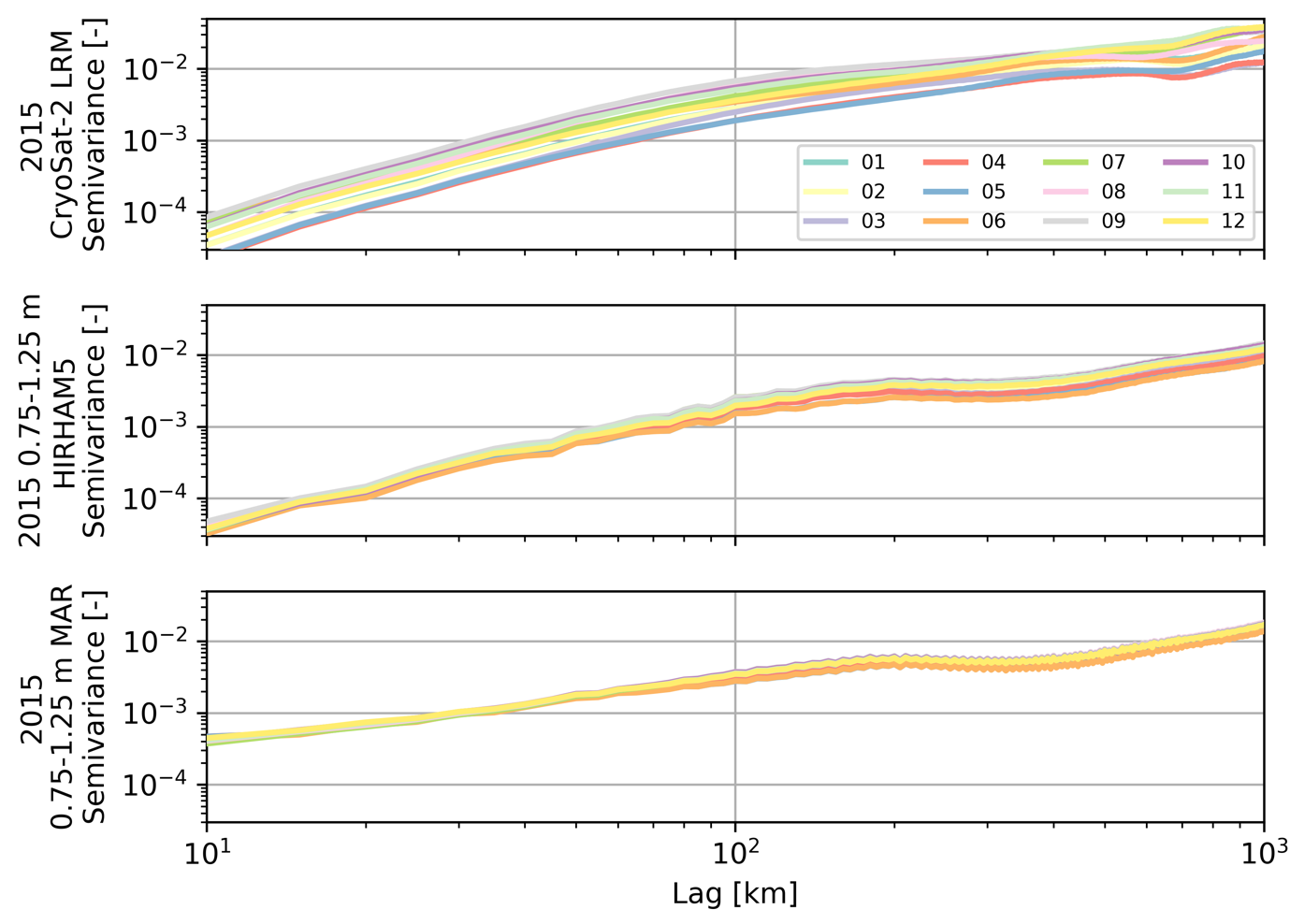


**Figure S19.** 2015 monthly empirical semivariograms derived from a) CryoSat-2 LRM densities as well as b) HIRHAM5 and c) MAR results within the CryoSat-2 LRM representative depth interval (0.75-1.25 m).


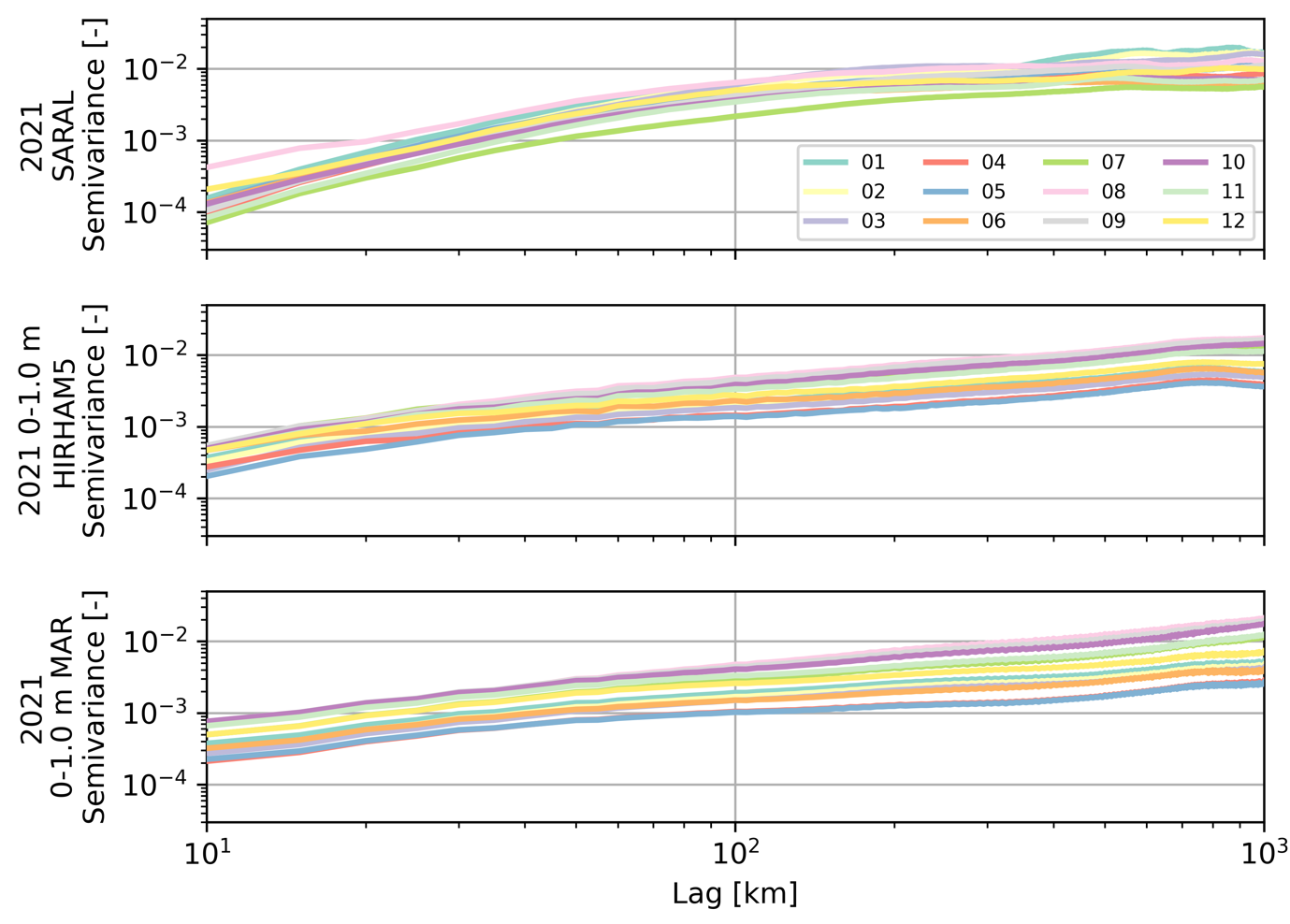


**Figure S20.** 2021 monthly empirical semivariograms derived from a) SARAL densities as well as b) HIRHAM5 and c) MAR results within the SARAL representative depth interval (0-1.0 m).


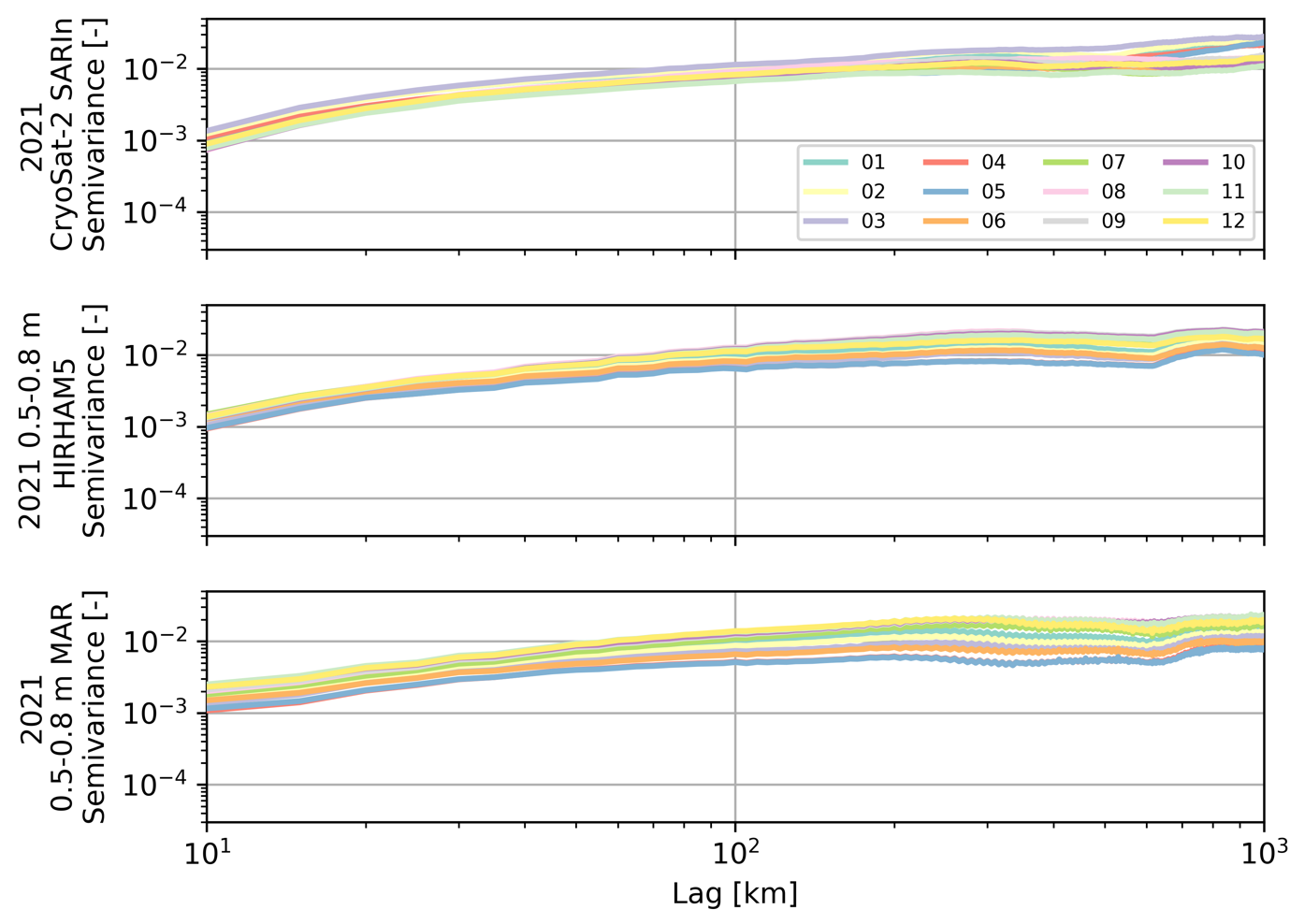


**Figure S21.** 2021 monthly empirical semivariograms derived from a) CryoSat-2 SARIn densities as well as b) HIRHAM5 and c) MAR results within the CryoSat-2 SARIn representative depth interval (0.5-0.8 m).


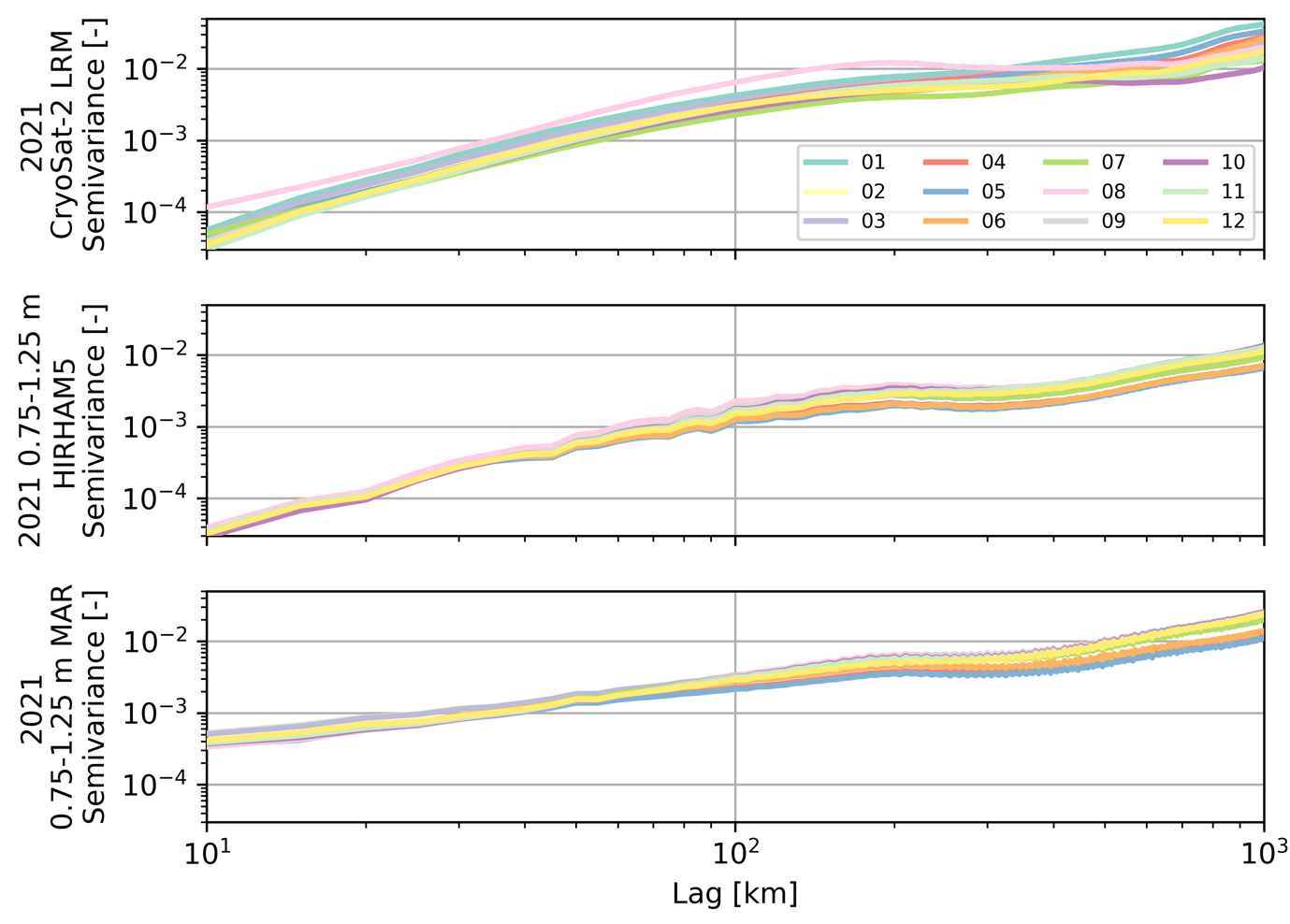


**Figure S22.** 2021 monthly empirical semivariograms derived from a) CryoSat-2 LRM densities as well as b) HIRHAM5 and c) MAR results within the CryoSat-2 LRM representative depth interval (0.75-1.25 m).
